# Supplementary material for: Confidence does not mediate a relationship between owner experience and likelihood of using weight management approaches for native ponies
Source: PLoS One. 2023 Oct 12;18(10):e0292886. doi: 10.1371/journal.pone.0292886 (PMC10569591; doi:10.1371/journal.pone.0292886)
Supplement: S1 File — (DOCX) [file pone.0292886.s001.docx]

Supplementary materials

Confidence does not mediate a relationship between owner experience and likelihood of using weight management approaches for native ponies

Ashley B. Ward ^1,2^*, Patricia A. Harris^3^, Caroline M. Argo^1^, Christine A. Watson^1^, Neil M. Burns^4^, Madalina Neacsu^2^, Wendy R. Russell ^2^, Dai Grove-White^5^, Philippa K. Morrison^1^

^1^ Scotland’s Rural College, Bucksburn, Aberdeen, UK

^2^ School of Medicine, Medical Sciences and Nutrition, The Rowett Institute, University of Aberdeen, Foresterhill, Aberdeen, UK

^3^ Equine Studies Group, Waltham Petcare Science Institute, Leicestershire, UK

^4^ Department of Rural Economy, Environment and Society, Scotland’s Rural College, Edinburgh, UK

^5^ Faculty of Health and Life Sciences, University of Liverpool, Wirral, UK

**S1 Table.** Univariate and multivariate GLMs explaining likelihood of undertaking weight management approaches as a function of confidence, and years of experience testing years of experience dichotomised at four different thresholds (4, 10, 20, or 30 years or over)

|  | <4 years vs ≥4 years' experience | | | <10 years vs ≥10 years' experience | | | <20 years vs ≥20 years' experience | | | <30 years vs ≥30 years' experience | | |
| --- | --- | --- | --- | --- | --- | --- | --- | --- | --- | --- | --- | --- |
|  | Univariate | Multivariate | | Univariate | Multivariate | | Univariate | Multivariate | | Univariate | Multivariate | |
|  | Experience | Confidence | Experience | Experience | Confidence | Experience | Experience | Confidence | Experience | Experience | Confidence | Experience |
| Monitor body condition (var 1) | p > 0.05 | p > 0.05 | p <0.001 *** | p > 0.05 | p > 0.05 | p > 0.05 | p > 0.05 | p <0.001 *** | p > 0.05 | p > 0.05 | p <0.001 *** | p > 0.05 |
| Monitor body condition (var 2) | p > 0.05 | p > 0.05 | p > 0.05 | p > 0.05 | p = 0.042 * | p > 0.05 | p > 0.05 | p = 0.039 * | p > 0.05 | p > 0.05 | p > 0.05 | p > 0.05 |
| Seasonal weight loss (var 1) | p > 0.05 | p > 0.05 | p > 0.05 | p > 0.05 | p > 0.05 | p > 0.05 | p > 0.05 | p > 0.05 | p > 0.05 | p > 0.05 | p > 0.05 | p > 0.05 |
| Seasonal weight loss (var 2) | p > 0.05 | p > 0.05 | p > 0.05 | p > 0.05 | p > 0.05 | p > 0.05 | p > 0.05 | p > 0.05 | p > 0.05 | p > 0.05 | p > 0.05 | p > 0.05 |
| Preserved forage (var 1) | p > 0.05 | p > 0.05 | p > 0.05 | p > 0.05 | p > 0.05 | p > 0.05 | p = 0.024 * | p = 0.040 * | p > 0.05 | p > 0.05 | p > 0.05 | p > 0.05 |
| Preserved forage (var 2) | p > 0.05 | p > 0.05 | p > 0.05 | p > 0.05 | p > 0.05 | p > 0.05 | p > 0.05 | p > 0.05 | p > 0.05 | p > 0.05 | p > 0.05 | p > 0.05 |
| Exercise | p > 0.05 | p > 0.05 | p > 0.05 | p > 0.05 | p > 0.05 | p > 0.05 | p = 0.016 * | p > 0.05 | p = 0.021 * | p > 0.05 | p > 0.05 | p > 0.05 |

**S2 Table. Additional information on respondent demographics and management practices.**

| Age group |  | |  |
| --- | --- | --- | --- |
| < 18 | 39 (7% [ 2.622, 11.215]) | |  |
| 18-25 | 84 (15% [10.490, 19.082]) | |  |
| 26-40 | 160 (28% [23.776, 32.369]) | |  |
| 41-60 | 243 (42% [38.287, 46.879]) | |  |
| > 60 | 46 (8% [ 3.846, 12.439]) | |  |
| Involvement in industry |  | | |
| Yes | 167 (29% [25.524, 33.067]) | | |
| No | 405 (71% [67.133, 74.675]) | | |
| Membership with equestrian organisation |  | | |
| Yes | 390 (68% [64.336, 72.105]) | | |
| No | 182 (32% [27.972, 35.741]) | | |
| Equestrian related qualifications |  | | |
| Yes | 170 (30% [26.049, 33.639]) | | |
| No | 402 (70% [66.608, 74.198]) | | |
| Frequency of weight monitoring |  | | |
| Weekly | 202 (35% [31.119, 39.761]) | | |
| Monthly | 155 (27% [22.902, 31.544]) | | |
| Occasionally | 104 (18% [13.986, 22.628]) | | |
| Daily | 89 (16% [11.364, 20.006]) | | |
| Annually | 22 (4% [<0.001, 8.292]) | | |
| Aware of preserved forage analysis |  |  |  |
| Yes | 466 (81% [78.497, 84.751]) |  |  |
| No | 106 (19% [15.559, 21.814]) |  |  |


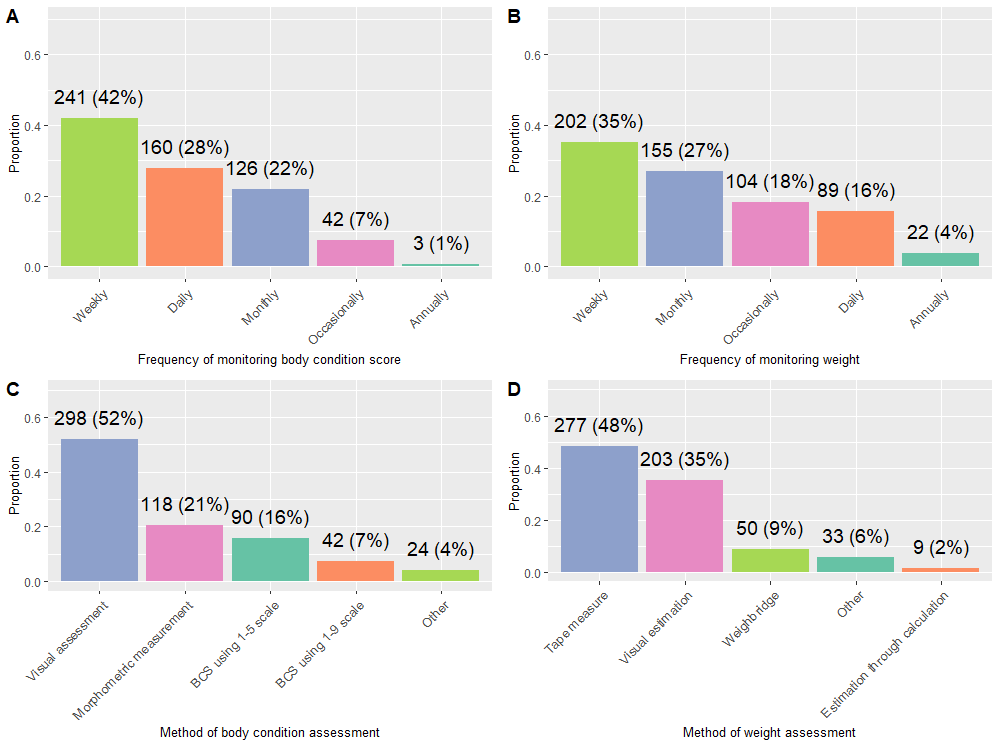


**Fig S1. Approaches to weight body condition monitoring and frequency of assessment.** (A) Frequency of body condition assessment and (B) body weight assessment, and methods used to assess (C) body condition and (D) body weight.

**
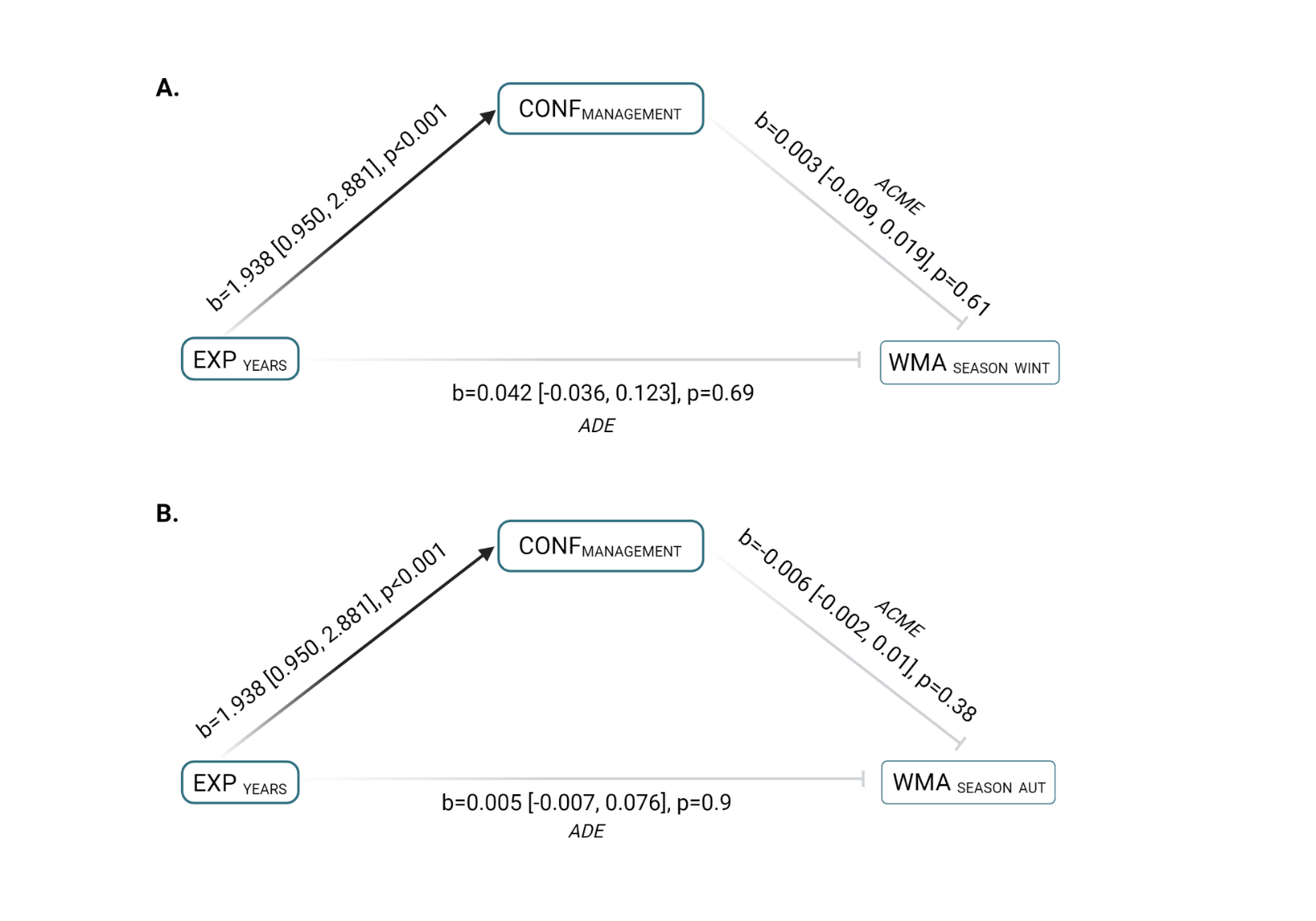
**

**Fig S2.** **Mediation model coefficients for the indirect effect (ACME) and average direct effect (ADE) of confidence (CONF _MANAGEMENT_) on the relationship between owners having >20 years’ experience caring for native ponies (EXP _YEARS_) and WMAs related to seasonal weight management.** (A) EXP _YEARS_ was significantly positively associated with CONF _MANAGEMENT,_ but this confidence did not mediate a relationship with promoting weight loss in winter (WMA _SEAS WINT_) (B) CONF _MANAGEMENT_ did not mediate a significant relationship between EXP _YEARS_ and promoting weight loss in autumn (WMA _SEAS AUT_). Figure created with Biorender.com.


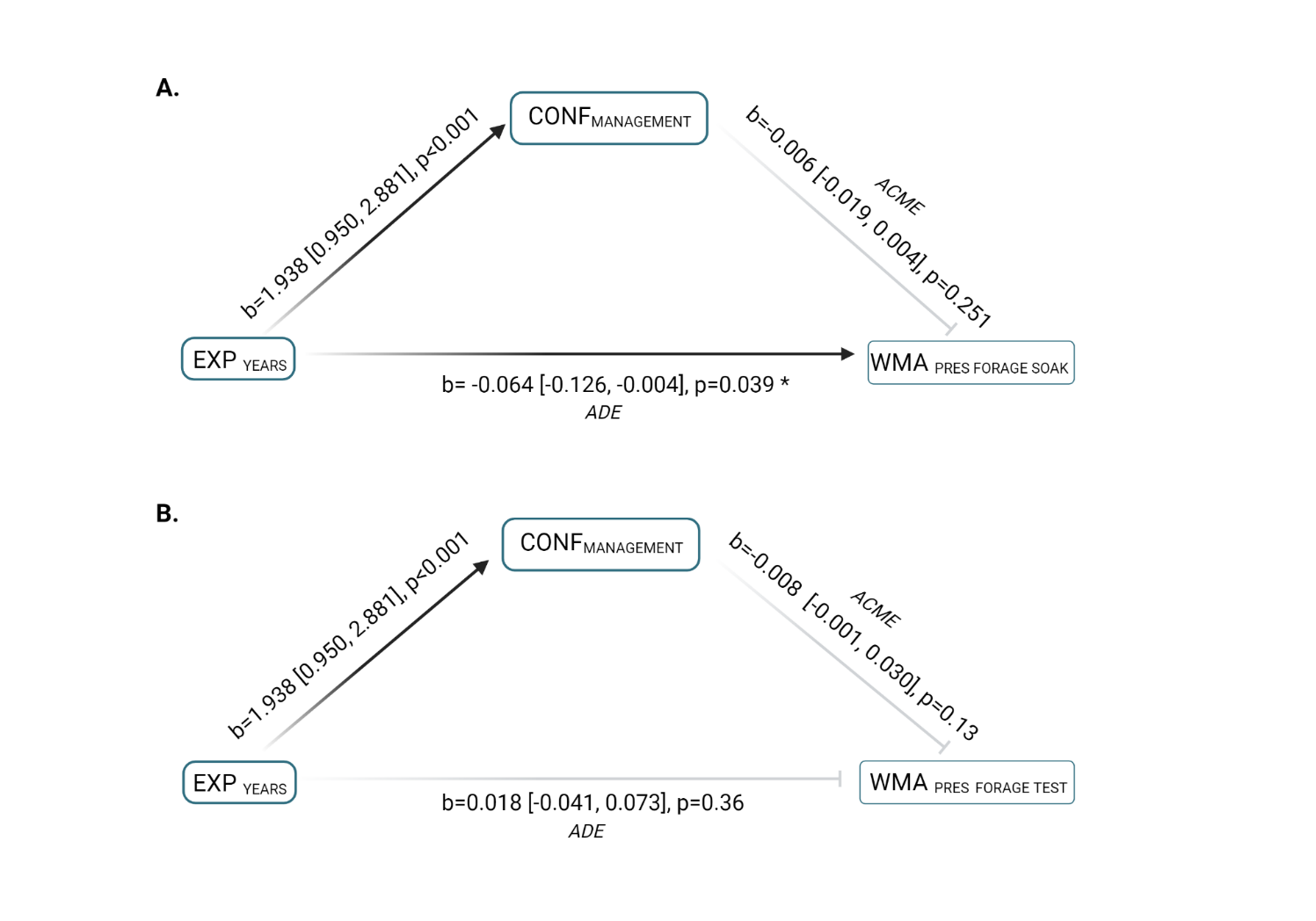


**Fig S3.** **Mediation model coefficients for the average causal mediation effect (ACME) and average direct effect (ADE) of confidence (CONF _MANAGEMENT_) on the relationship between owners having >20 years’ experience caring for native ponies (EXP _YEARS_) and their likelihood of using WMAs related to preserved forage.** (A) EXP _YEARS_ was significantly positively associated with CONF _MANAGEMENT_, but confidence did not mediate a significant relationship with providing soaked hay (WMA _PRES_ _FORAGE SOAK_) (B) CONF _MANAGEMENT_ did not mediate a significant relationship between EXP _YEARS_ and analysing preserved forage (WMA _PRES_ _FORAGE TEST_). Figure created with Biorender.com.
