# Supplementary material for: Confidence does not mediate a relationship between owner experience and likelihood of using weight management approaches for native ponies
Source: PLoS One. 2023 Oct 12;18(10):e0292886. doi: 10.1371/journal.pone.0292886 (PMC10569591; doi:10.1371/journal.pone.0292886)
Supplement: S2 File — (DOCX) [file pone.0292886.s002.docx]

Supplementary materials

**Confidence does not mediate a relationship between owner experience and likelihood of using weight management approaches for native ponies**

A.B. Ward ^1,2^, P.A. Harris^3^, C.M. Argo^1^, C. Watson^1^, N.M. Burns^4^, M. Neacsu^2^, W. Russell ^2^, D. Grove-White^5^, P.K. Morrison*^1^

^1^ Scotland’s Rural College, Bucksburn, Aberdeen, UK

^2^ School of Medicine, Medical Sciences and Nutrition, The Rowett Institute, University of Aberdeen, Foresterhill, Aberdeen, UK

^3^ Equine Studies Group, Waltham Petcare Science Institute, Leicestershire, UK

^4^ Department of Rural Economy, Environment and Society, Scotland’s Rural College, Edinburgh, UK

^5^ Faculty of Health and Life Sciences, University of Liverpool, Wirral, UK

Survey questions

Where in Scotland are your horses based? (Please provide a postcode area)

How many horses do you currently own or care for in total? (This includes horses that you own, loan, share, lease or have rescued)

Is the horse or pony that you care for considered to be a "native breed - type"?

Please specify your age range:

Other than owning / sharing / loaning horses, do you have an additional involvement in the equine industry?

Which of the following best describes your involvement in the equine industry? (Select all that apply)

What is your profession?

For how many years have you been making decisions about horse management?

Are you currently a member of any equestrian organisations / societies?

Are you currently a member of any of the following organisations? (Select all that apply)

Have you completed, or are you currently undertaking, any equestrian or veterinary industry-based qualifications?

Which of the following qualifications do you hold or currently work towards? (Select all that apply)

How frequently do you make decisions about the following aspects of horse management?

-Pasture management (e.g. topping, rolling, fertilising fields)

-Herd management

-Choosing the time spent grazing / turnout routine

-Choosing the type / quality of preserved forage to feed

-Choosing the amount of preserved forage to feed

-Deciding on the type of complementary feed

-Deciding on the amount of complementary feed

-Rugging, clipping, stable bandaging

How confident do you feel in making decisions regarding the following aspects of horse management?

-Pasture turnout routine

-Herd management

-Time spent grazing / turnout routine

-Preserved forage type / quantity

-Preserved forage amount

-Type of complementary feed

-Amount of complementary feed

-Rugging, clipping, stable bandaging

From which of the following sources do you prefer to seek advice from in regard to:

-Your horse's health

-Your horse's nutrition

-Your horse's pasture management (e.g. topping, rolling, fertilising fields)

-Your horse's weight

Do you monitor your horse's body condition (how frequently)?

In general, how do you monitor your horses' body condition?

Have you ever been formally shown how to carry out body condition scoring on horses?

How did you learn this skill ?

Has your horse's body condition score been measured by a veterinarian or nutritionist whilst being under your care?

Do you monitor your horse's weight (how frequently)?

In general, how do you monitor your horse's weight?

With respect to season, when managing a native-type pony, which of the following would you prefer to do:

Aim to maintain weight

Aim to promote weight loss

Aim to promote weight gain

In terms of the facilities and equipment available to you, do you face any issues when managing your horse's weight? (Please select all that apply).

Do you feed your horses preserved forage in addition to, or to replace, their grazing across the various seasons?

-Spring/Summer

-Autumn/ Winter

Do any of the following clinical conditions or dietary needs determine the preserved forage you feed to your horses? (Please select all that apply).

Which preserved forage source would you prefer to feed your horses?

Is this the same as the preserved forage your horses are fed?

Has there been an analysis of the nutritional content of your hay/haylage?

Were you aware that analysis could be carried out on hay/haylage?

When was the last time that your horses hay/haylage was analysed?

What was your hay/haylage analysed for?

Were you advised to have your hay or haylage analysed?

Please specify who advised you to do this, and why.

Do you currently feed your horses a feed balancer?

What are your main reasons for feeding a balancer? (Please select all that apply)

We often receive advice from multiple sources on how to feed our horses. From the following sources of feeding advice, please select the ONE which has had the greatest influence on your decision making regarding feeding your horse supplementary concentrates/ hard feed?

In general, what do you feel is the most important reason for exercising your horse(s)?

With respect to exercising your horse, please select the greatest barrier that you face:

On a scale from 1 to 5, please indicate how confident you are in recognising the signs of the following conditions in horses:

-Laminitis

-Obesity

-Equine metabolic syndrome

-Colic

-PPID (Equine Cushing’s Disease)

-Loss of condition

Have you ever identified laminitis in a horse or pony?

From which of the following sources have you obtained the most guidance on how to manage laminitis?

Please rank the following interventions in order of how confident you would be in delivering these to manage a laminitic horse:

-Provision of comfortable bedding

-Provision of appropriate dietary changes

-Pain management

-Rehabilitation (e.g. building up turnout / fitness)

In regard to managing laminitis, are there any subjects that you would like more information about? (Please select all that apply)

What behaviour or signs would indicate to you that a horse or pony had laminitis?

From where did you hear about this survey?
