# Supplementary material for: Confidence does not mediate a relationship between owner experience and likelihood of using weight management approaches for native ponies
Source: PLoS One. 2023 Oct 12;18(10):e0292886. doi: 10.1371/journal.pone.0292886 (PMC10569591; doi:10.1371/journal.pone.0292886)
Supplement: Attachment — Submitted filename: PONE-D-23-11189 (1)Optimized_Optimized_Optimized_Optimized_Optimized.pdf [file pone.0292886.s003.pdf]

PLOS ONE

Confidence does not mediate a relationship between owner experience and likelihood of using weight management approaches for native ponies

--Manuscript Draft--

**Manuscript Number:**	PONE-D-23-11189
**Article Type:**	Research Article
**Full Title:**	Confidence does not mediate a relationship between owner experience and likelihood of using weight management approaches for native ponies
**Short Title:**	Confidence does not influence the relationship between pony owner experience and weight management approach
**Corresponding Author:**	Ashley Blair Ward, BSc<br>Scotland's Rural College<br>Aberdeen, Aberdeen UNITED KINGDOM
**Keywords:**	Equine; Laminitis; Equine Obesity; Nutrition; Owner confidence; Mediation analysis; Native pony; Weight management; Equine metabolic disease; Hay soaking; Forage Analysis; Survey, Questionnaire, Cross-sectional survey; Horse owner.
**Abstract:**	Laminitis is a painful condition which manifests in the hoof and has significant welfare implications for affected horses and ponies. Native ponies are at increased risk of obesity and metabolic perturbations, such as insulin dysregulation (ID), associated with laminitis risk. Management and feeding practices can be adapted to maintain healthy body condition and support metabolic health, but owners may inadvertently provide their ponies with inappropriate management leading to obesity and exacerbating laminitis risk. Adoption of preventative weight management approaches (WMAs), including regular monitoring of body condition, providing appropriate preserved forage, promoting seasonal weight loss, and using exercise accordingly, are key in supporting native ponies' metabolic health. The factors influencing the adoption of WMAs, such as owners' experience and confidence, require exploration. The aim of the current study was to understand factors influencing owners' likelihood to undertake certain WMAs, to develop our understanding of suitable intervention targets. A total of 571 responses to an online cross-sectional questionnaire were analysed. Mediation analysis revealed that whilst long term (>20 years) experience caring for native ponies was associated with owners increased, self-reported confidence in identifying disease and managing their native ponies, this did not translate to an increased likelihood of implementing WMAs. Conversely, respondents who managed ponies with metabolic dietary requirements (i.e., obesity, laminitis, or equine metabolic syndrome) were more likely to use WMAs related to feeding, seasonal weight management and exercise. Owner confidence was assessed and rejected as a mediator of the relationship between experience and WMA use. These results highlight the need for further work that elucidates the pathways leading owners to undertake action against obesity without the need for ponies to develop overt disease, as well as suggesting a need for long term managers of native ponies to update management practices with preventative care as the focus.
**Order of Authors:**	Ashley Blair Ward, BSc
	Patricia A Harris, MA, PhD, VetMB, DipECVCN, MRCVS
	Caroline M Argo, BVSc, BSc (Hons), PhD, DipECAR, MRCVS
	Christine A Watson, BSc (Hons), PhD
	Neil M Burns, BSc (Hons), PGCert, MSc, PhD
	Madalina Neacsu, Dipl Eng, MSc, PhD
	Wendy R Russell, BSc (Hons), PhD, CSci, CChem, MRSC
	Dai Grove-White, BVSc, MSc, DBR, PhD, DECBHM, FRCVS
	Philippa K Morrison, BSc(Hons), PhD, FHEA
**Opposed Reviewers:**	

Powered by Editorial Manager® and ProduXion Manager® from Aries Systems Corporation

Additional Information:	
**Question**	**Response**
**Financial Disclosure**<br><br>Enter a financial disclosure statement that describes the sources of funding for the work included in this submission. Review the submission guidelines for detailed requirements. View published research articles from *PLOS ONE* for specific examples.<br><br>This statement is required for submission and **will appear in the published article** if the submission is accepted. Please make sure it is accurate.<br><br>**Unfunded studies**<br>Enter: *The author(s) received no specific funding for this work.*<br><br>**Funded studies**<br>Enter a statement with the following details:<br>• Initials of the authors who received each award<br>• Grant numbers awarded to each author<br>• The full name of each funder<br>• URL of each funder website<br>• Did the sponsors or funders play any role in the study design, data collection and analysis, decision to publish, or preparation of the manuscript?<br>• **NO** - Include this sentence at the end of your statement: *The funders had no role in study design, data collection and analysis, decision to publish, or preparation of the manuscript.*<br>• **YES** - Specify the role(s) played.<br><br>* typeset	This study was funded by Mars Petcare and is part of a PhD studentship funded by the Scottish Funding Council Research Excellence Grant (REG). Authors WR and MN receive salary support from the Rural and Environment Science and Analytical Services Division (RESAS). With the exception of PH (employed by the funding organization), the funding organization did not have any additional role in the conceptualization, methodology, investigation, data curation, formal analysis, decision to publish, or preparation of the manuscript. PH was involved in study design, data interpretation, and manuscript preparation.
**Competing Interests**<br><br>Use the instructions below to enter a competing interest statement for this submission. On behalf of all authors, disclose any competing interests that could be perceived to bias this work—acknowledging all financial support and any other relevant financial or non-financial competing interests.	Co-author PH is employed by the funding organization. This does not alter our adherence to PLOS ONE policies on sharing data and materials. All other authors declare that they have no competing interests.

Powered by Editorial Manager® and ProduXion Manager® from Aries Systems Corporation

This statement is required for submission and **will appear in the published article** if the submission is accepted. Please make sure it is accurate and that any funding sources listed in your Funding Information later in the submission form are also declared in your Financial Disclosure statement.<br><br>View published research articles from *PLOS ONE* for specific examples.<br><br>**NO authors have competing interests**<br><br>Enter: *The authors have declared that no competing interests exist.*<br><br>**Authors with competing interests**<br><br>Enter competing interest details beginning with this statement:<br><br>*I have read the journal's policy and the authors of this manuscript have the following competing interests: [insert competing interests here]*<br><br>* typeset	
**Ethics Statement**<br><br>Enter an ethics statement for this submission. This statement is required if the study involved:<br><br>• Human participants<br>• Human specimens or tissue<br>• Vertebrate animals or cephalopods<br>• Vertebrate embryos or tissues<br>• Field research<br><br>Write "N/A" if the submission does not require an ethics statement.<br><br>General guidance is provided below. Consult the submission guidelines for detailed instructions. **Make sure that all information entered here is included in the**	Ethical approval for the study was obtained from SRUCs Social Science ethics committee on the 30th of January 2020. Participant consent was obtained through an online tick box procedure within the questionnaire.

**Methods section of the manuscript.**<br><br>**Format for specific study types**<br><br>**Human Subject Research (involving human participants and/or tissue)**<br>• Give the name of the institutional review board or ethics committee that approved the study<br>• Include the approval number and/or a statement indicating approval of this research<br>• Indicate the form of consent obtained (written/oral) or the reason that consent was not obtained (e.g. the data were analyzed anonymously)<br><br>**Animal Research (involving vertebrate animals, embryos or tissues)**<br>• Provide the name of the Institutional Animal Care and Use Committee (IACUC) or other relevant ethics board that reviewed the study protocol, and indicate whether they approved this research or granted a formal waiver of ethical approval<br>• Include an approval number if one was obtained<br>• If the study involved *non-human primates*, add *additional details* about animal welfare and steps taken to ameliorate suffering<br>• If anesthesia, euthanasia, or any kind of animal sacrifice is part of the study, include briefly which substances and/or methods were applied<br><br>**Field Research**<br><br>Include the following details if this study involves the collection of plant, animal, or other materials from a natural setting:<br>• Field permit number<br>• Name of the institution or relevant body that granted permission	
**Data Availability**<br><br>Authors are required to make all data underlying the findings described fully available, without restriction, and from the time of publication. PLOS allows rare exceptions to address legal and ethical concerns. See the PLOS Data Policy and FAQ for detailed information.	Yes - all data are fully available without restriction

Powered by Editorial Manager® and ProduXion Manager® from Aries Systems Corporation

A Data Availability Statement describing where the data can be found is required at submission. Your answers to this question constitute the Data Availability Statement and **will be published in the article**, if accepted.<br><br>**Important:** Stating 'data available on request from the author' is not sufficient. If your data are only available upon request, select 'No' for the first question and explain your exceptional situation in the text box.<br><br>Do the authors confirm that all data underlying the findings described in their manuscript are fully available without restriction?	
**Describe where the data may be found in full sentences. If you are copying our sample text, replace any instances of XXX with the appropriate details.**<br><br>• If the data are **held or will be held in a public repository**, include URLs, accession numbers or DOIs. If this information will only be available after acceptance, indicate this by ticking the box below. For example: *All XXX files are available from the XXX database (accession number(s) XXX, XXX.*).<br>• If the data are all contained **within the manuscript and/or Supporting Information files**, enter the following: *All relevant data are within the manuscript and its Supporting Information files.*<br>• If neither of these applies but you are able to provide **details of access elsewhere**, with or without limitations, please do so. For example:<br><br>*Data cannot be shared publicly because of [XXX]. Data are available from the XXX Institutional Data Access / Ethics Committee (contact via XXX) for researchers who meet the criteria for access to confidential data.*<br><br>*The data underlying the results*	All data files are available from SRUC's FigShare repository (10.58073/SRUC.22608412).

presented in the study are available from (include the name of the third party and contact information or URL).<br>• This text is appropriate if the data are owned by a third party and authors do not have permission to share the data.<br><br>* typeset	
Additional data availability information:	Tick here if the URLs/accession numbers/DOIs will be available only after acceptance of the manuscript for publication so that we can ensure their inclusion before publication.

Powered by Editorial Manager® and ProduXion Manager® from Aries Systems Corporation

13th April 2023

Ms Ashley Ward
Department of Veterinary & Animal Sciences
Aberdeen
AB21 9YA
Scotland

+44 (0)7714069044
Ashley.ward@sruc.ac.uk
www.sruc.ac.uk

Dear Editor,

We would be grateful if you could accept the attached original research article for consideration for publication as an original article. The manuscript is entitled,

"Confidence does not mediate a relationship between owner experience and likelihood of using weight management approaches for native ponies"

by, Ashley Ward, Patricia Harris, Caroline Argo, Christine Watson, Neil Burns, Madalina Neacsu, Wendy Russell, Dai Grove-White, and Philippa Morrison

Much like other companion animals, equids in the UK face a worsening obesity epidemic. This is particularly true for leisure equids, of which a popular breed are native ponies. British native ponies are at increased risk of obesity, and of developing associated metabolic diseases such as insulin dysregulation and laminitis. Management strategies that limit ponies' intake of high concentrations of non-structural carbohydrates and enable the rapid detection of excess adipose tissue deposition, should be incorporated into the standard management of native ponies as preventative measures. However, measures of the current level of uptake of these practices, and the factors influencing the likelihood of owners using them, are currently unexplored.

In the present paper we explore the usage of evidence-based weight management approaches (WMAs) by native pony owners across Scotland. We test a theoretical model that owner experience influences the likelihood of using these practices, and that this relationship is mediated by confidence. Through assessment of this model, we address a key question of whether improving owner confidence is a suitable target for intervention for behaviour change in native pony owners.

According to the findings of the presented sample, long term experience was significantly associated with confidence, but not with WMA usage. Meanwhile, we found that the opposite was true for current experience of managing a pony with metabolic disease (either laminitis, equine metabolic syndrome, or overweight). This measure of experience was significantly associated an increased likelihood of undertaking beneficial management practices in feeding and promoting metabolic health, but had no association with confidence. Owner confidence did not appear to mediate a relationship between experience and likelihood of WMA usage, suggesting that it may be an ineffective target for behaviour change intervention in this field.

We believe that these findings will be of interest to your journal's readership. We certify that this paper consists of original, unpublished work which is not under consideration for publication elsewhere. We know of no conflicts of interest associated with this publication, and there has been no significant financial support for this work that could have influenced its outcome. As Corresponding Author, I confirm that the manuscript has been read and approved for submission by the named authors. We suggest that appropriate Academic Editors to handle the manuscript may include: Agricola Odi, Sherin Reda Rouby or Csaba Varga.

We hope that our manuscript will reach the high-quality standards for publication in PlosOne and we look forward to receiving your response.

Yours Sincerely,

Ashley Ward (corresponding author)

At the heart of the natural economy

This and other documents can be made available in other formats such as large print and/or community languages on request. If you would like a translated copy, please contact us with the details of the format/language required.

SRUC is a charitable company limited by guarantee, Scottish Charity Number: SC003712. Registered in Scotland, Company Number: SCO1033046. Registered Office: Peter Wilson Building, King's Buildings, West Mains Road, Edinburgh EH9 3JG. SAC Commercial Limited. An SRUC Company. Registered in Scotland, Company Number SC148684. Registered Office: Peter Wilson Building, King's Buildings, West Mains Road, Edinburgh, EH9 3JG.

Manuscript

Click here to access/download;Manuscript;Ward_et_al_2023_manuscript.do

Confidence does not mediate a relationship between owner experience and likelihood of using weight management approaches for native ponies

Ashley B. Ward [1,2]*, Patricia A. Harris[3], Caroline M. Argo[1], Christine A. Watson[1], Neil M. Burns[4], Madalina Neacsu[2], Wendy R. Russell [2], Dai Grove-White[5], Philippa K. Morrison[1]

[1] Scotland's Rural College, Bucksburn, Aberdeen, UK

[2] School of Medicine, Medical Sciences and Nutrition, The Rowett Institute, University of Aberdeen, Foresterhill, Aberdeen, UK

[3] Equine Studies Group, Waltham Petcare Science Institute, Leicestershire, UK

[4] Department of Rural Economy, Environment and Society, Scotland's Rural College, Edinburgh, UK

[5] Faculty of Health and Life Sciences, University of Liverpool, Wirral, UK

*Corresponding Author

Email: Ashley.Ward@sruc.ac.uk (AW)

Abstract

Laminitis is a painful condition which manifests in the hoof and has significant welfare implications for affected horses and ponies. Native ponies are at increased risk of obesity and metabolic perturbations, such as insulin dysregulation (ID), associated with laminitis risk. Management and feeding practices can be adapted to maintain healthy body condition and support metabolic health, but owners may inadvertently provide their ponies with inappropriate management leading to obesity and exacerbating laminitis risk. Adoption of preventative weight management approaches (WMAs), including regular monitoring of body condition, providing appropriate preserved forage, promoting seasonal weight loss, and using exercise accordingly, are key in supporting native ponies' metabolic health. The factors influencing the adoption of WMAs, such as owners' experience and confidence, require exploration. The aim of the current study was to understand factors influencing owners' likelihood to undertake certain WMAs, to develop our understanding of suitable intervention targets. A total of 571 responses to an online cross-sectional questionnaire were analysed. Mediation analysis revealed that whilst long term (>20 years) experience caring for native ponies was associated with owners increased, self-reported confidence in identifying disease and managing their native ponies, this did not translate to an increased likelihood of implementing WMAs. Conversely, respondents who managed ponies with metabolic dietary requirements (i.e., obesity, laminitis, or equine metabolic syndrome) were more likely to use WMAs related to feeding, seasonal weight management and exercise. Owner confidence was assessed and rejected as a mediator of the relationship between experience and WMA use. These results highlight the need for further work that elucidates the pathways leading owners to undertake action against obesity without the need for ponies to develop overt disease, as well as suggesting a need for long term managers of native ponies to update management practices with preventative care as the focus.

Introduction

Obesity continues to dominate discussions around current equine welfare issues [1,2]. Between 21% and 35% of the UK's leisure horses and ponies are thought to be obese [3–5], although higher prevalence has been reported in native breed-type ponies [6] who are at increased risk of obesity[5]. In addition to obesity, British native ponies are also at an increased risk of developing insulin dysregulation (ID) [7,8] and laminitis [9]. Although obesity is a key risk factor, lean animals can be insulin dysregulated [7,10], highlighting the complex interactions between these metabolic conditions which are in turn linked to an increased risk of endocrinopathic laminitis and its recurrence [6,7,11]. British native ponies are therefore an important target for obesity interventions to improve equine welfare in the UK.

Weight management approaches (WMAs) that help induce weight loss and improve insulin sensitivity in the horse have been extensively researched. Forms of WMAs include dietary restriction and provision of forages that are low in Non-Structural Carbohydrates (NSCs), which can induce body mass losses in ponies under controlled study conditions [12–15]. Of particular importance for native ponies, whose metabolism adaptively slows during colder seasons to conserve energy, are dietary restriction and low NSC carbohydrate feeding which can be used as a WMA to replicate the natural reductions in available nutrients during cold seasons [16]. Incorporating exercise into diet-restricted regimens may also have beneficial effects upon insulin sensitivity [17], thus reducing laminitis risk.

Positive results of equine weight-loss interventions have been demonstrated in the field [18], and scientific findings have been translated into freely available guides that are offered by various organisations, including the Blue Cross [19], The Horse Trust [20], and World Horse Welfare [21]. These weight management guides describe WMAs in categories relating to monitoring body condition, providing appropriate feeding, promoting seasonal weight loss, providing the appropriate exercise, and controlling access to pasture. Widespread use of proposed WMAs could help to reduce obesity across the leisure horse population. However, the success of equine weight loss intervention is largely determined by owners' compliance [18], along with the horses' access to pasture.

Owner compliance with weight-loss regimens and adherence to recommended WMAs may be influenced by a combination of behavioural, social, and physical factors [22]. Recreational horse owner beliefs' on the appropriate management for their animals appears to be linked to knowledge and experience [23], both of which are modifiable factors, suggesting a role for targeted education and training to promote the best practices for native pony weight management. Effective behavioural intervention through education requires that the target group identify their own requirement for such training, and research regarding horse owners' identification of obesity in their own horses has shown that many owners may have difficulty doing this [24,25]. Attitudes driven by subjective knowledge gained in the equestrian field, such as holding differing thresholds for acceptable body condition based on equestrian discipline [26], and the mistrust of practices involving dietary restriction [25], may involve learned behaviours nested in tradition which no longer serve the modern management of native ponies.

Confidence in abilities drawn from subjective experience could make owners less likely to seek or accept changing approaches to management. At its most extreme, evidence of this behavioural phenomenon can be seen in relation to the COVID-19 pandemic, where subjective and biased information sourcing appeared to be linked with scepticism towards governmental health recommendations [27]. In the present context, non-evidence-based knowledge acquisition leading to inappropriate native pony management could be linked with a worsening obesity problem for UK leisure equids. Understanding how owner experience influences confidence, and how this confidence in turn is associated with the implementation of WMAs, could reveal insights into the drivers of owner behaviour in relation to promoting a healthy weight in their animals

The aim of the present study was to explore the role played by owner experience and confidence in integrating evidence-based WMAs into the management of their native breed-type ponies. This was addressed by the development of a causal model of owners' experience and their undertaking of WMAs in four areas relating to weight management, including: monitoring body condition, providing appropriate feeding, promoting seasonal weight loss, and promoting exercise. Subsequently, mediation analysis was employed to evaluate the direct effect of native pony owners experience upon

their likelihood of using WMAs, and the indirect effect of experience upon WMA usage via confidence.

Methods

1.1. Questionnaire design

A cross-sectional survey was designed to collect information on all aspects of the general management of native ponies in Scotland. Ethical approval for the study was obtained from SRUCs Social Science ethics committee on the 30th of January 2020. The survey was made available for data collection immediately after ethical approval was obtained until the 13th of October 2021. The 77-item questionnaire comprised eight areas of questioning: personal experience, general management, weight management, feeding and nutrition, exercise, and laminitis care. The entire survey included a variety of binary, Likert scale items, multiple response, ranking and open text formats. The present analysis focused on binary variables relating to experience and WMAs and 5-point Likert scales related to confidence. Survey questions (excluding those related to pasture management which were outside the scope of the present study) can be found in the Supplementary Materials.

1.2. Data collection

The anonymised online survey was created using Smart Survey software and was promoted by press release and social media activity over an 18-month period from . Respondents were entered into a prize draw to win a 100 GBP gift voucher as an incentive to undertake the survey. Upon survey closure, one respondent was selected at random for receipt of this incentive prize, after which point identifiable data were permanently deleted. Respondents identified as residing outside of Scotland or those that did not own and care for native ponies were excluded from further analysis based on answers to two screening questions.

1.3. Data preparation

Years of experience was converted to a binary variable by defining owners with less than or greater than 20 years of experience (EXP_{YEARS}). The cut off for experience was selected at 20 years

because it was considered that these groups effectively represented owners with long term experience caring for native ponies (>20 years), and those within a range of experience from limited to reasonable (<20 years). The resulting binary categories preserved the higher proportion owners at the upper extreme whilst providing a suitable proportion of owners with fewer years of experience to allow for fair comparison.

Owners were also categorized based on their response to a question asking which clinical conditions or dietary needs influenced their feeding practices. A new binary variable was created to categorize owners who self-reported managing their ponies' nutrition for metabolic disease-related conditions, compared to those who did not (EXP_{MET}). Dichotomisation was achieved by assigning "Yes" to owners who indicated any of laminitis, equine metabolic syndrome (EMS) or weight management as conditions that influenced their horses' feeding, and "No" to those who did not select these options. Questions relating to WMAs were presented to respondents in a nominal, single choice format. These variables were then converted to binary variables to allow comparison between those who undertook a given weight management approach and those who did not. The original nominal variable categories are summarised along with the final binary versions in the results.

Eight Likert Scale items each on a 5-point scale were used to measure owners' self-reported confidence in various aspects of their ponies' management, and six items measured owners' self-reported confidence in identifying the signs of health conditions affecting the horse. For mediation analysis, the Likert items were summed to create two latent continuous Likert Scale variables. The first latent variable represented "Confidence in management" ($CONF_{MANAGEMENT}$); the sum of 8 Likert items relating to management of native ponies. The second latent variable represented "Confidence in identifying signs of disease" ($CONF_{DISEASE}$); the sum of 6 Likert items relating to owners' confidence in identifying the signs of obesity, laminitis, EMS, pituitary pars intermedia dysfunction (PPID), colic and body condition loss. Composite scores of the latent Likert Scales were assumed to represent the underlying continuous distribution of confidence.

Initial data screening was performed in Excel version 2301. Data re-coding, formatting, visualisation, and analysis were conducted using R version 4.2 [28]. The full code used for analysis can be viewed in the supporting information (Supporting Information S1).

1.4. Data analysis

Descriptive statistics were performed with proportions and 95% confidence interval approximation to summarise categorical variables [29]. Cronbach's Alpha statistic was calculated as a measure of similarity between the scale items and was used to evaluate whether the items of scales relating to the two latent variables, namely CONF $_{MANAGEMENT}$ and CONF $_{DISEASE}$ reflected the same respective constructs. Skewedness and kurtosis of these continuous latent variables was assessed. Both variables demonstrated right skew, and so to minimise the impact of this violation of the normality assumption, statistical inference was made from confidence intervals derived from 5000 bootstrap samples [30].

Mediation analysis was performed using the R package mediation [31]. Models were constructed to simultaneously assess the direct effect of the independent variable (IV) "EXP" on the dependent variable (DV) likelihood of WMAs, and the indirect effect of the IV on the DV through the latent mediator "Conf"[32]. Outcome models were fitted using binary probit GLMs, and the mediator models were fitted via linear regression of the effect of experience on the latent scales of confidence. Bias-corrected bootstrap-based confidence intervals were used examine the indirect effects of experience on likelihood of undertaking WMAs via confidence. Fig 1 shows a directional acyclic graph outlining the shared structure of the models outlined.

Fig 1. Directional acyclic graph demonstrating mediation model structures. The effects of mediation on the relationships between explanatory and outcome variables were evaluated simultaneously through estimating combined associations between experience (EXP) and mediator, and between mediator and weight management approach (WMA). Mediation was assessed through evaluating the indirect effect (pathway c`)[32] and comparing the strength and direction of this effect relative to the average direct effect (pathway c). Dark lines with arrow heads indicate significant relationships, whilst grey lines with blunt ends indicate non-significant relationships. Figure created with Biorender.com.

Relationships between binary and categorical variables assessed outside of mediation analysis were modelled via generalised linear regression using a logit link function, or using loglinear models where multiple tests of independence were made. Results for regressions are reported with beta coefficients (b), 95% confidence intervals (95% CIs) and p-values (p).

Results

The survey received a total of 1779 responses. All rows (responses) with missing data for questions included in the analysis were excluded, leaving a final sample size of n=571. The sample was comprised largely of leisure owners (71%) as opposed to industry professionals, and the majority of the sample respondents did not hold industry related qualifications (70%). Roughly half (53%) of owners managed the dietary requirements of a pony with any one of laminitis, EMS, or overweight, and almost half (47%) had over 20 years of experience managing native ponies. A full summary of sample can be seen in Supplementary Table S1 and Fig S1. See Table 1 for summary statistics pertinent to the proceeding analysis.

Table 1. Descriptive statistics of respondent demographics and equestrian experience.

Variable	Proportion of total (n=571) (n (% [95% CI]))
Years of caring for native ponies	
0-3	41 (7% [2.972, 11.534])
4-9	103 (18% [13.811, 22.373])
10-19	161(28% [23.951, 35.513])
20-29	117 (20% [16.259, 24.821])
>30	150 (26% [22.080, 30.590])
**All >20 years (EXP $_{YEARS}$)**	**267 (47%)**
*Feeding ponies for specific health condition	
Laminitis	218 (38% [35.00, 43.626])
Weight management	145 (25% [21.903, 30.520])
Equine metabolic syndrome	86 (15% [11.311, 19.928])
Equine gastric ulcer syndrome	36 (6% [0.023, 0.110[illegible]
Respiratory condition	36 (6% [0.023, 0.110])
Colic	36 (6% [0.023, 0.110])
**Managing any of EMS/laminitis/weight (EXP $_{MET}$)**	**305 (53%)**
Frequency of body condition assessment	
Daily	160 (28% [23.601, 32.367])
Weekly	241 (42% [37.762, 46.528])
Monthly	126 (22% [17.657, 26.423])
Annually	3 (1% [0, 4.919])
Occasionally	42 (7% [2.972, 11.738])
Never	0
**More frequent than annual assessment (WMA $_{BCS\ FREQ}$)**	**526 (92%)**
*Method used for body condition assessment	

Visual assessment	298 (52% [48.077, 56.430])
Morphometric measurement	118 (21% [16.608, 24.961])
BCS (1-5 scale)	90 (16% [11.714, 20.066])
BCS (1-9 scale)	42 (7% [3.322, 11.674])
Other	24 (4% [0.175, 8.528])
**Standardised method (WMA $_{\text{BCS METHOD}}$)**	**250 (44%)**
Analysed preserved forage	
**Yes (WMA $_{\text{PRES FORAGE TEST}}$)**	**74 (13%** [9.266, 16.910])
No	338 (68% [64.161, 71.805])
Unknown	110 (19% [15.559, 23.204])
Preserved forage	
Hay	275 (48% [44.056, 52.402])
Mixture of hay and haylage	98 (17% [13.112, 21.458])
**Soaked hay (WMA $_{\text{PRES FORAGE SOAK}}$)**	**94 (16% [12.413, 20.759])**
Haylage	68 (12% [7.867, 16.213])
Steamed hay	22 (4% [<0.001, 8.171])
Short chop fibre source	9 (2% [<0.001, 5.899])
None	6 (1% [0.001, 5.374])
Weight management approach (Autumn)	
Maintain	371 (64% [61.014, 68.949])
**Encourage loss (WMA $_{\text{SEASON AUT}}$)**	**153 (27% [22.902, 30.837])**
Encourage gain	48 (8% [4.545, 12.481])
Weight management approach (Winter)	
Maintain	330 (58% [523.671, 62.057])
**Encourage loss (WMA $_{\text{SEASON WINT}}$)**	**201 (35% [31.119, 39.504])**
Encourage gain	41 (7% [3.147, 11.532])
Most important aspect of exercising pony	
Horses' performance	172 (30% [25.350, 33.933])
Personal enjoyment	169 (30% [25.874, 34.458])
**Weight management (WMA $_{\text{EXERCISE}}$)**	104 (18% [13.986, 22.570])
Boredom relief (horses')	88 (15% [11.189, 19.773])
Other	28 (5% [0.699, 9.283]).
Not applicable to my horses	11 (2% [0.000, 6.311])

* Multiple response question (the sum of proportions >1.0)
[i] Variables in bold are included in mediation analysis

In order of most frequent to least, owners reported being "Very" confident in; pasture management (61%), rugging/ clipping/ stabling (60%), controlling access to pasture (56%), herd management (54%), providing the appropriate type of preserved forage (50%), providing the appropriate type of complementary feeding (50%), providing the appropriate amount of supplementary feeding (48%), and providing the appropriate amount of preserved forage (48%) (Fig 2). Owner confidence in identifying signs of disease was more variable that confidence in management practices. The highest confidence rating was most often indicated for identifying; obesity (72%), loss of condition (65%), laminitis (59%), colic (58%), PPID (26%), and EMS (20%).

Respondents expressed markedly lower confidence in identifying the signs of EMS and PPID in comparison to identifying the signs of other conditions, and any management practices (Fig 2).

Fig 2. Percentage of respondents self-reported confidence level in ability to perform management of native ponies, and to identify signs of disease. Owners ranked Likert style items on a scale of 1-5 with 1 being "not confident at all" and 5 being "very confident". Bars to the left of the central line include 50% of neutral responses and negative scores (e.g. not confident), and to the right of the central line include 50% of neutral responses and positive scores (e.g. fairly or very confident). Plot generated using R package HH [33].

Latent confidence variables formed from the sum of categorical responses demonstrated left skew toward the higher confidence (Table 2). This skew was present in both measures of confidence but more so for confidence in management (CONF $_{MANAGEMENT}$), which had a median score more than double that of confidence in identifying signs of disease (CONF $_{DISEASE}$). Cronbach's alpha for CONF $_{MANAGEMENT}$ and CONF $_{DISEASE}$ were 0.948, and 0.745 respectively, indicating "excellent", and "acceptable" levels of similarity amongst scale items (based on an arbitrary scale of Cronbach's alpha statistic meaningfulness [34] to represent respective underlying concepts.

Table 2. Summary of the Likert scales of owners' confidence in managing their native ponies, and confidence in identifying the signs of disease.

	Median; Q1-Q3[a]; Min-max	Skewedness	Kurtosis	Cronbach's Alpha
CONF $_{MANAGEMENT}$ (Range: 8-40)	36; 32-40; 8-40	-1.540	2.393	0.948
CONF $_{DISEASE}$ (Range: 6- 30)	17; 15-19; 4-20	-1.352	2.662	0.745

[a] Q1 – 1ST quartile, Q3- 3rd quartile.

Neither CONF $_{DISEASE}$ nor CONF $_{MANAGEMENT}$ were associated with caring for a pony with metabolic disease related dietary requirements (EXP $_{MET}$), but both were significantly associated with having >20 years of experience managing native ponies (EXP $_{YEARS}$) (Table 3). Due to the lack of

association between EXP_{MET} and either confidence variable, mediation of the relationship between this measure of experience and the likelihood of undertaking the WMAs was rejected.

Table 3. Relationships between confidence scales and the independent experience variables.

	$\text{EXP}_{\text{YEARS}}$		EXP_{MET}	
Outcome	B (p-value)	95% CIs	B (p-value)	95% CIs
$\text{CONF}_{\text{MANAGEMENT}}$	1.145 (<0.001)	0.674, 1.615	0.053 (0.870)	-0.407, 0.527
$\text{CONF}_{\text{DISEASE}}$	1.938 (<0.001)	0.950, 2.881	-0.570 (0.254)	-1.541, 0.427

WMA 1: Monitoring body condition

Monitoring body condition was performed by respondents at various frequencies, most commonly on a weekly basis (42%), followed by monitoring daily (28%), monthly (22%), and occasionally or annually (8%) (Table 1). Assessment of their ponies' body weight was made less frequently; weekly assessment was reported by 32%, and 6% of owners had never assessed body weight. Standardised assessment of body weight was defined as involving a calibrated weighbridge, or morphometric measurement of some part of the horses' body. Standardised assessment of body condition was defined as involving the use of either a six-point (0-5) scale [35] or a nine-point (1-9) scale [36] or using morphometric measurements combined with the use of an online body condition calculator tool. Roughly half of owners used standardised methods to estimate their ponies' weight (49%) which was similar to the proportion who used standardised methods to assess body condition (42%). Less frequent assessment of bodyweight was undertaken by owners adopting a standardised method as opposed to a visual assessment ($b=-0.392$, 95% CI [-0.130, -0.656], $p=0.003$), in contrast to frequency of body condition scoring which was not significantly influenced by whether a standardised or visual method was used ($b=0.227$, 95% CI [-0.160, 0.621], $p=0.255$).

Neither EXP_{MET} or $\text{EXP}_{\text{YEARS}}$, were significantly directly associated with the likelihood of owners using more frequent ($\text{WMA}_{\text{BCS FREQ}}$) or standardised body condition assessment ($\text{WMA}_{\text{BCS METHOD}}$) (Table 4).

Table 4. Direct relationships between experience WMAs related to body condition monitoring.

	EXP $_{YEARS}$		EXP $_{MET}$	
Outcome	B (p-value)	95% CIs	B (p-value)	95% CIs
WMA $_{BCS\ FREQ}$	-0.093(0.543)	-0.395, 0.209	0.141 (0.358)	-0.160, 0.444
WMA $_{BCS\ METHOD}$	-0.169(0.111)	-0.383, 0.032	0.121(0.277)	-0.087, 0.328

Mediation by CONF $_{DISEASE}$, was assessed for the relationship between EXP $_{YEARS}$ and WMA $_{BCS\ FREQ}$, and the bootstrapped unstandardised indirect effect coefficient was significant (Fig 3A). The total effect of direct and indirect pathways and the average direct effect were negative, but not statistically significant. The indirect effect between EXP $_{YEARS}$ and WMA $_{BCS\ METHOD}$ via CONF $_{DISEASE}$ was statistically significant with a positive coefficient (Fig 3B).

Fig 3. Mediation model coefficients for the indirect effect (ACME) and average direct effect (ADE) of confidence on the relationship between owners having >20 years' experience caring for native ponies (EXP $_{YEARS}$) and WMAs related to monitoring body condition.
(A) EXP $_{YEARS}$ was significantly positively associated with confidence in identifying signs of disease (CONF $_{DISEASE}$). The ACME was significant and positive and ADE was negative and non-significant, demonstrating conflicting effects of the positive indirect pathway between EXP $_{YEARS}$ upon likelihood of more frequent body condition assessment (WMA BCS $_{FREQ}$) via CONF $_{DISEASE}$, and the negative average direct effects of EXP $_{YEARS}$ upon WMA BCS $_{FREQ}$ (**B**) Coefficients between EXP $_{YEARS}$ and owners' likelihood of using standardised methods of body condition assessment WMA BCS $_{METHOD}$ also demonstrated competing direct (negative) and indirect (positive) effects, the ADE was again not significant. Figure created with Biorender.com.

WMA 2: Seasonal weight management

As opposed to aiming for weight loss or gain, the majority of respondents aimed to "maintain" their horses' weight over the autumn (65%), winter (58%), spring (54%), and summer (55%) months. Weight management approach was dependent upon season (X^2 (df)=57.35(6), p <0.001). Encouraging

weight gain and aiming to maintain weight were associated with the autumn months (September, October, November), more so than other seasons. Promoting weight loss was associated with the Spring and Summer months (March – August) (Fig 4).

Fig 4. Weight management approach according to season. Rectangle height represents relative frequencies of each combination of weight management approach and season. Shading indicates the pattern of residuals from a loglinear model of weight management approach predicted by season, where intensity of shading represents magnitude, blue colour indicates a positive relationship, and pink colour indicates a negative relationship.

As previously indicated (Table 3), the latent $CONF_{MANAGEMENT}$ measure was significantly associated with EXP_{YEARS} but not with EXP_{MET}, and so mediation by confidence was not tested for the relationship between EXP_{MET} and WMAs relating to seasonal management. Independently of confidence, EXP_{MET} was associated with a significantly greater likelihood of promoting weight loss (opposed to promoting weight gain or maintenance) across autumn ($WMA_{SEASON\ AUT}$) and winter ($WMA_{SEASON\ WINT}$) (Table 5).

Table 5. Direct relationships between experience and WMAs relating to seasonal weight management of the horse.

	EXP_{YEARS}		EXP_{MET}	
Outcome	B (p-value)	95% CIs	B (p-value)	95% CIs
$WMA_{SEASON\ WINT}$	0.117 (0.255)	-0.095, 0.329	0.364 (0.001)	0.150, 0.579
$WMA_{SEASON\ AUT}$	$-0.002e^{-1}$ (0.998)	-0.218, 0.224	0.525 (<0.001)	0.298, 0.754

While EXP_{YEARS} was associated with $CONF_{MANAGEMENT}$ (Table 2), it was not associated with using the WMAs in seasonal management (Table 4). There was no evidence to suggest that confidence mediated a relationship between EXP_{YEARS} and promoting weight loss in the winter (Fig 5A) or autumn (Fig 5B).

Fig 5. Mediation model coefficients for the indirect effect (ACME) and average direct effect (ADE) of confidence ($\text{CONF}_{\text{MANAGEMENT}}$) on the relationship between owners having >20 years' experience caring for native ponies ($\text{EXP}_{\text{YEARS}}$) and WMAs related to seasonal weight management.

(A) $\text{EXP}_{\text{YEARS}}$ was significantly positively associated with $\text{CONF}_{\text{MANAGEMENT,}}$ but this confidence did not mediate a relationship with promoting weight loss in winter ($\text{WMA}_{\text{SEAS WINT}}$) (B) $\text{CONF}_{\text{MANAGEMENT}}$ did not mediate a significant relationship between $\text{EXP}_{\text{YEARS}}$ and promoting weight loss in autumn ($\text{WMA}_{\text{SEAS AUT}}$). Figure created with Biorender.com.

WMA 3: Providing appropriate preserved forage

Between March and August, 56% of owners added preserved forage to their native ponies' diet to supplement their grazing. Provision of preserved forage increased to 95% during the autumn and winter months from September to February. Roughly half of respondents provided their ponies with a vitamin and mineral feed balancer (55%), with the most common reason being to supplement a forage-based diet (38%). Of the 267 owners with >20 years of experience, 38 had undertaken preserved forage analysis compared to 36 of 304 owners with < 20 years' experience. Probit regression showed that $\text{EXP}_{\text{YEARS}}$ was significantly associated with a reduced likelihood of undertaking preserved forage analysis, and EXP_{MET} was significantly positively associated with both preserved forage analysis and hay soaking (Table 6).

Table 6. Direct relationships between experience and likelihood of undertaking WMAs related to providing appropriate preserved forage.

	$\text{EXP}_{\text{YEARS}}$		EXP_{MET}	
Outcome	B (p-value)	95% CIs	B (p-value)	95% CIs
$\text{WMA}_{\text{PRES FORAGE TEST}}$	-0.286 (0.024)	-0.538, -0.036	0.502 (<0.001)	0.247, 0.763
$\text{WMA}_{\text{PRES FORAGE SOAK}}$	0.115 (0.397)	-0.146, 0.377	0.291 (0.032)	0.026, 0.561

Mediation analysis showed that the indirect path between EXP $_{\text{YEARS}}$ and WMA $_{\text{PRES FORAGE TEST}}$ was not significant, providing no evidence to suggest that CONF$_{\text{MANAGEMENT}}$ significantly influenced a relationship between the two variables (Fig 6A). The likelihood of owners undertaking preserved forage analysis was also not affected either directly or indirectly through increased confidence, by years of experience (Fig 6B).

Fig 6. Mediation model coefficients for the average causal mediation effect (ACME) and average direct effect (ADE) of confidence (CONF $_{\text{MANAGEMENT}}$) on the relationship between owners having >20 years' experience caring for native ponies (EXP $_{\text{YEARS}}$) and their likelihood of using WMAs related to preserved forage.

(A) EXP $_{\text{YEARS}}$ was significantly positively associated with CONF $_{\text{MANAGEMENT}}$, but confidence did not mediate a significant relationship with providing soaked hay (WMA $_{\text{PRES FORAGE SOAK}}$) (B) CONF $_{\text{MANAGEMENT}}$ did not mediate a significant relationship between EXP $_{\text{YEARS}}$ and analysing preserved forage (WMA $_{\text{PRES FORAGE TEST}}$). Figure created with Biorender.com.

WMA 4: Exercise

The aspect of exercising ponies that was most frequently selected as being "most important" was the horse's performance, followed by personal enjoyment (Table 1). Eighteen percent of owners felt that weight management was the most important aspect of exercising their ponies. The likelihood of selecting this choice was significantly positively associated with EXP $_{\text{MET}}$, while being significantly negatively related to EXP $_{\text{YEARS}}$ (Table 7).

Table 7. Association between experience variables and the likelihood of perceiving weight management as the most important outcome of exercising ponies.

	EXP $_{\text{YEARS}}$		EXP $_{\text{MET}}$	
Outcome	B (p-value)	95% CIs	B (p-value)	95% CIs
WMA $_{\text{EXERCISE}}$	-0.297 (0.016)	-0.542, -0.054	0.683 (<0.001)	0.428, 0.945

The indirect effect of the relationship between EXP $_{\text{YEARS}}$ and WMA $_{\text{EXERCISE}}$ was not significant, thus there was no evidence to suggest mediation by CONF $_{\text{DIS}}$ (Fig 7).

Fig 7. Mediation model coefficients for the average causal mediation effect (ACME) and average direct effect (ADE) of confidence in ability to identify disease (CONF $_{DIS}$) on the relationship between owners having >20 years' experience caring for native ponies EXP $_{YEARS}$ and their likelihood of perceiving weight management as the most important outcome of exercising their ponies (WMA $_{EXERCISE}$). CONF $_{DIS}$ did not mediate a significant relationship between EXP $_{YEARS}$ and WMA $_{EXERCISE.}$ Figure created with Biorender.com.

Discussion

In addition to its association with laminitis and metabolic disease, obesity is a significant welfare issue facing native ponies- with reproductive, orthopaedic and cardiovascular implications [1]. Reducing the prevalence of equine obesity and related conditions requires a better understanding of the human behaviour underpinning horse care. Despite increased attention to the prevalence and risks of obesity, studies of UK horses have shown no drastic decline in its prevalence [3–6,37]. Previous work has highlighted the need for research to explore causal pathways driving attitudinal and behavioural factors influencing horse owner decision making [23].

The current study found evidence to suggest that having over 20 years of experience managing native ponies leads to increased owner confidence in management and disease identification in their native ponies, but this confidence did not significantly influence the likelihood of those owners' undertaking a selection of weight management approaches (WMAs). Independently of confidence, years of experience had a negative direct effect on owners' likelihood of undertaking WMAs related to monitoring ponies' body condition, seasonal weight management, forage provision and exercise. In contrast, owners undertaking the nutritional management of metabolic disease related conditions in their ponies were no more or less likely to have greater confidence in either management or disease recognition. However, this group of owners were more likely to undertake positive weight management approaches for their ponies in areas related to seasonal weight management, preserved

forage provision, and exercise. These findings are consistent with previously stated impressions [38] that management-based intervention is more likely to be employed by owners after the development of disease -where it would be more beneficial in a preventative capacity.

In the present study, owners demonstrated high self-reported confidence in their ability to identify obesity in their native ponies, with confidence levels significantly greater in those with over 20 years of experience in native pony management. Confidence in identifying the signs of obesity, as for laminitis, have been equally high in previous reports [38]. Compared with their confidence in identifying colic, loss of condition and obesity, owners tend to have lower self-reported confidence [38], as well as weaker knowledge [39], in equine metabolic syndrome (EMS). The present work found owners' confidence in identifying laminitis to be more consistent with their confidence in identifying obesity, loss of condition and colic than with confidence in identifying metabolic diseases such as EMS and pituitary pars intermedia dysfunction (PPID). Together with research which found that that only 1 in 5 surveyed horse owners were familiar with EMS [39], these results could indicate a lack of understanding amongst owners of the clinical importance of obesity and laminitis and their potential roles as risk factors for complex metabolic disease.

Confusion in the horse owning community around EMS could reflect the evolving clinical definition of EMS, which recognises the potential for ID and laminitis in to occur non-obese animals [40], although obesity when present may exacerbate clinical outcomes [41]. Clarity in owner education around equine metabolism and breed specific management may improve knowledge of the associated risk of laminitis that increases with obesity and insulin dysregulation. This then begs the question as to whether improving owner understanding of equine metabolic disease would in turn lead to improved health outcomes for the native ponies under their care. Quantifying owner understanding was out-with the scope of the present study, and it cannot be assumed that more years of experience equates to greater knowledge, however it seems plausible that greater understanding would in turn lead to greater confidence. Findings of the present study suggested that in most cases, confidence did not mediate a relationship between experience and routine use of WMAs, and therefore may not be

the appropriate behavioural target for intervention in owners' management of ponies at risk of obesity and laminitis.

The high self-reported confidence in obesity recognition seen presently may have been misplaced, given the high prevalence of obesity in the leisure horse demographic, and known tendency of owners towards weight underestimation in cases of obesity [3,24,26,42]. Instead, a shift toward undertaking some of the WMAs surveyed was more likely where owners had direct experience of managing ponies with metabolic dietary requirements. These findings align with previous work which found that owners engaging in proactive weight management had often experienced a "trigger" event, such as their horse developing laminitis, or a veterinarian pointing out their horses' obesity, prompting them to undertake concerted weight management efforts [25]. Further, research has shown owners to perceive metabolic disease, such as EMS, as having a strong impact upon equine welfare, which may result in a reciprocal negative impact on their own emotional well-being where their own animals have the condition [39]. This evidence seems to support the development of interventions that emphasize the "real-life" impact of managing metabolic disease in ponies and highlight the practical and welfare-based benefits (cost, time, improvement in the animals' long-term quality of life) that disease prevention could afford.

Neither experience, managing a pony with metabolic dietary requirements, nor owner confidence in identifying obesity in their ponies influenced the method or frequency of body condition assessment. Whilst monitoring equine bodyweight and/ or body condition in general was used by the majority of respondents - similar to the proportions found previously [43] - a large proportion of owners used visual, subjective methods. This is in accordance with work showing that owners preferred to adopt visual assessment of their horse's body condition [38]. Owner monitoring of body condition is of vital importance to complement veterinary interventions for equine obesity [1,2], but the continued reliance on non-standardised approaches (e.g. visual assessment) highlights the potential to further promote this aspect of preventative management for native ponies. The frequent use of standardised methods, such as Henneke's 9-point body condition scoring scale [36], may aid the detection of weight gain and help owners to develop familiarity with the physical assessment of adiposity. Such assessment is particularly important for detecting gradual, seasonal fat

accumulation which can increase ponies' risk of laminitis and obesity [9,16]. Although non-significant, our results showed a trend where higher confidence increased the likelihood of owners' using WMAs related to monitoring body condition, which was in contrast to the decreased likelihood of doing so that was directly associated with more years of experience. It may be useful to highlight to owners the need to "update" skills around disease recognition in their ponies to enable them to make timely management adjustments that promote their pony's wellbeing. This might work towards combating a reduced likelihood of undertaking certain WMAs as years of experience in the industry accumulate. As changes in body condition occur slowly, the recommended approach would be the combined use of body condition scoring and morphometric measurements, which may give owners greater incentive to consistently monitor their ponies body condition and weight.

The presence of strong seasonal changes in appetite and basal energy requirements within our native breed ponies means they are not suited to modern husbandry methods that can provide year-round access to high quality forages [16]. These seasonal changes in metabolism provide an ideal scenario to support body mass losses during the winter months in the obese animal. There was a higher likelihood of adopting a weight loss approach over the autumn and winter by the owners of ponies with metabolic dietary requirements, indicating an awareness by owners that such management may be beneficial for weight and metabolic disease management. This study also provided evidence of owners using management approaches that could obstruct the natural variations in equine bodyweight [44], where they aimed to maintain their horses' weight across all seasons. Without physical assessment, it is not possible to identify whether owners were maintaining their ponies at a healthy weight. However, extrapolation of previous estimates of obesity in a pony population [6] suggests that in up to 70% of cases, owners could have been aiming to maintain their ponies in overweight or even obese states.

The WMAs relating to preserved forage provision were more likely to be undertaken by owners actively managing ponies with metabolic dietary requirements. Current recommendations suggest that owners should undertake analysis of forage for non-structural and water- soluble carbohydrate content (NSC and WSC respectively) alongside soaking to provide information on the suitability of the forage for the horse [45]. Research has identified a high degree of variability in preserved forage analysis

[46], indicating that multiple samples are required for the most representative results. This, combined with the fact that livery yards frequently change sources of preserved forage due to high usage, may deter owners from undertaking forage analysis. With regard to hay soaking, its efficacy in reducing sugar content of preserved forage is dependent upon soaking conditions [47,48], and owners may be physically unable to adopt this practice due to its physically demanding and time consuming nature [22]. Despite their limitations, hay soaking and forage analysis are measures which could encourage consideration of the NSC concentrations in preserved forage by owners making decisions around providing the appropriate nutrition for their ponies. Whilst not likely to prevent metabolic disease, when used pre-emptively these practices could reduce the risk of exacerbating diagnosed or sub-clinical metabolic disorders through inadvertent provision of high NSC forages [49]. Limited reporting of the rates of forage analysis undertaken makes it difficult to identify whether the 14% usage found in the present study is comparable to the true proportion of native pony owners using forage analysis in the population. However, taken with the finding that 80% of respondents were aware of the practice, it appears that the promotion of forage analysis as a useful tool to aid the management of ponies at risk of metabolic disease may be necessary.

Perception of the value of exercise in the management of metabolic conditions in the horse may have been more positive in owners with experience of the benefits it can confer to horse health. Those managing ponies with EMS, laminitis or overweight were more likely to perceive weight management as the most important outcome of exercise. Combined with dietary restriction, low-intensity exercise can improve insulin sensitivity in obese horses [17], and recent work has highlighted a potentially protective role of exercise against laminitis risk [11]. Given that acutely laminitic animals should not be exercised, the emphasis of this practice in prevention is key. Information regarding ponies' intended purpose was not collected, however, native breed types are typically popular for leisure, breeding and / or competition purposes and our results showed that the horses' performance, and owners' personal enjoyment were the favoured outcomes of exercise. As this was a subjective question without a single preferred answer, the intention was not to suggest that weight management was the most important aspect of exercising ponies. Through asking owners to select the most important aspect from their perspective, the question provided insight on owner priorities in terms of

exercising their ponies. The results indicate that the intended purpose of the horse could take precedence over the horses' nutritional and energy-based requirements, leading owners to perceive the weight management aspect of exercise as less important than other areas. Promoting the benefits of incorporating exercise into the management of non-laminitic ponies could help to reduce those ponies' risk of metabolic disease and may encourage a positive change in the perception of exercise as a tool for preventative management.

Ideally, management of native ponies would undergo an overhaul in terms of standard practice, with laminitis prevention at the forefront of decision making. Changing human behaviour is the key to achieving animal welfare goals which depend upon human management of nutrition. Within the adapted "Ten Tasks" framework of human behaviour change proposed for meaningful improvements in animal welfare [50,51], the present work contributes towards understanding the mechanisms relating to horse owner experience and confidence that may help in sustaining current management practices used for native pony management . The proceeding steps towards reducing obesity, laminitis and ID in UK equids are to construct behavioural models specific to the equine management, and progress in this area has already been made. Comprehensive behavioural analysis of the human role in equine obesity has shown the complex social environment within the horse management domain [22], and suggests that interventions are targeted not only toward owner knowledge of obesity and its devastating consequences for horses, but also toward owner motivation and equestrian social structures. The present findings are in agreement with the concept that generic approaches to improving confidence in weight management are not sufficient. Instead, work towards integrating WMAs, like the 9-point body condition scoring system, forage analysis, exercise, and utilising winter metabolism, into the common vernacular of native pony care could help the shift owner perceptions of obesity, weight management and laminitis prevention.

As for any questionnaire, there is inherent respondent bias that must be acknowledged. In the current study, participants were an experienced group, and a prior interest in laminitis and/or native pony management could have influenced owners' decision to undertake the survey. The present study focused on owners of native ponies in Scotland, and so generalisation of the results to non-native horse care, or to populations outside of Scotland, may be limited. Further, an effect of survival bias

may influence the higher likelihood of undertaking WMAs by the owners of native ponies with metabolic disease associated dietary requirements, whose animals may be less likely to have survived had they not altered their management. The mediation analysis adopted in this study is a novel approach to providing key insights into the causal pathways between predictors and outcomes. Mediation analysis has previously been utilised in the field of canine veterinary medicine as a tool for understanding the role of caregiver burden the relationship between clinical signs of osteoarthritis, and euthanasia [52]. The application of structural equation modelling associated multivariate techniques which can be used to test causal pathways will be an important tool for future research that seeks to understand more about the human factors related to equine disease. Proposed model structures are theoretical but are designed with prior knowledge. There is a risk that theoretical models do not explain the true relationship between the factors assessed, and in testing for causation, there is a risk of masking true effects and relationships which exist outside of our pre-defined structure [53]. Care was taken to consider paths and assess potential confounding relationships, and the limitations of quantitative surveys in analysis of human behaviour are acknowledged.

Conclusion

The results presented suggest that whilst confidence in care may increase with years of experience, it does not appear to drive the use of management practices that support risk reduction for laminitis, obesity, and EMS. In native pony management, WMAs may be underutilised as a preventative approach, instead being more commonly undertaken by owners after their pony has developed a metabolic health condition of concern. Obesity and laminitis may be perceived by native pony owners as distinct from metabolic disease, demonstrating the need to highlight to owners the interconnected nature of these conditions, and the seriousness of their impact upon pony welfare.

Acknowledgments

We wish to gratefully acknowledge the horse owners who took the time to undertake this survey.

References

1. Shepherd M, Harris P, Martinson KL. Nutritional Considerations When Dealing with an Obese Adult Equine. Vet Clin N Am - Equine Pract. 2021;37: 111–137. doi:10.1016/j.cveq.2020.12.004

2. Rendle D, McGregor Argo C, Bowen M, Carslake H, German A, Harris P, et al. Equine obesity: current perspectives. UK-Vet Equine. 2018;2: 1–19. doi:10.12968/ukve.2018.2.s2.3

3. Stephenson HM, Green MJ, Freeman SL. Short communications: Prevalence of obesity in a population of horses in the UK. Vet Rec. 2011;168. doi:10.1136/vr.c6281

4. Robin CA, Ireland JL, Wylie CE, Collins SN, Verheyen KLP, Newton JR. Prevalence of and risk factors for equine obesity in Great Britain based on owner-reported body condition scores. Equine Vet J. 2015;47: 196–201. doi:10.1111/evj.12275

5. Giles SL, Rands SA, Nicol CJ, Harris PA. Obesity prevalence and associated risk factors in outdoor living domestic horses and ponies. PeerJ. 2014;2: e299. doi:10.7717/peerj.299

6. Menzies-Gow NJ, Harris PA, Elliott J. Prospective cohort study evaluating risk factors for the development of pasture-associated laminitis in the United Kingdom. Equine Vet J. 2017;49: 300–306. doi:10.1111/evj.12606

7. Box JR, McGowan CM, Raekallio MR, Mykkänen AK, Carslake H, Karikoski NP. Insulin dysregulation in a population of Finnhorses and associated phenotypic markers of obesity. J Vet Intern Med. 2020;34: 1599–1605. doi:10.1111/jvim.15782

8. Carslake HB, Pinchbeck GL, McGowan CM. Equine metabolic syndrome in UK native ponies and cobs is highly prevalent with modifiable risk factors. Equine Vet J. 2021;53: 923–934. doi:10.1111/evj.13378

9. Pollard D, Wylie CE, Verheyen KLP, Newton JR. Identification of modifiable factors associated with owner-reported equine laminitis in Britain using a web-based cohort study approach. BMC Vet Res. 2019;15. doi:10.1186/s12917-019-1798-8

10. Luthersson N, Mannfalk M, Parkin TDH, Harris P. Laminitis: Risk Factors and Outcome in a Group of Danish Horses. J Equine Vet Sci. 2017;53: 68–73. doi:10.1016/j.jevs.2016.03.006

11. Knowles EJ, Elliott J, Harris PA, Chang YM, Menzies-Gow NJ. Predictors of laminitis development in a cohort of nonlaminitic ponies. Equine Vet J. 2022. doi:10.1111/evj.13572

12. McGowan CM, Dugdale AH, Pinchbeck GL, Argo CMG. Dietary restriction in combination with a nutraceutical supplement for the management of equine metabolic syndrome in horses. Vet J. 2013;196: 153–159. doi:10.1016/j.tvjl.2012.10.007

13. Dugdale AHA, Curtis GC, Cripps P, Harris PA, Argo MM. Effect of dietary restriction on body condition, composition and welfare of overweight and obese pony mares. Equine Vet J. 2010;42: 600–610. doi:10.1111/j.2042-3306.2010.00110.x

14. Morrison PK, Newbold CJ, Jones E, Worgan HJ, Grove-White DH, Dugdale AH, et al. The equine gastrointestinal microbiome: Impacts of weight-loss. BMC Vet Res. 2020;16. doi:10.1186/s12917-020-02295-6

15. Dosi MCM, Kirton R, Hallsworth S, Keen JA, Morgan RA. Inducing weight loss in native ponies: Is straw a viable alternative to hay? Vet Rec. 2020;187. doi:10.1136/vr.105793

16. Dugdale AHA, Curtis GC, Cripps PJ, Harris PA, Argo CMG. Effects of season and body condition on appetite, body mass and body composition in ad libitum fed pony mares. Vet J. 2011;190: 329–337. doi:10.1016/j.tvjl.2010.11.009

17. Bamford NJ, Potter SJ, Baskerville CL, Harris PA, Bailey SR. Influence of dietary restriction and low-intensity exercise on weight loss and insulin sensitivity in obese equids. J Vet Intern Med. 2019;33: 280–286. doi:10.1111/jvim.15374

18. Gill JC, Pratt-Phillips SE, Mansmann R, Siciliano PD. Weight Loss Management in Client-Owned Horses. J Equine Vet Sci. 2016;39: 80–89. doi:10.1016/j.jevs.2015.12.014

19. Fat Horse Slim A practical guide for managing weight loss in horses. Available: https://www.bluecross.org.uk/fat-horse-slim

20. Tamzin Furtado, Elizabeth Perkins, Gina Pinchbeck, Cathy McGowan, Francine Watkins, Rob Christley. When the grass is greener: The Equine Weight Management guide for every horse, every yard, and every owner. Available: https://www.liverpool.ac.uk/media/livacuk/equine/documents/Equine,Weight,Management.pdf

21. World Horse Welfare. Is your horse the Right Weight? Available: https://www.worldhorsewelfare.org/advice/management/right-weight

22. Furtado T, Perkins E, Pinchbeck G, McGowan C, Watkins F, Christley R. Exploring human behavior change in equine welfare: Insights from a COM-B analysis of the UK's equine obesity epidemic. 2022 pp. 01–13.

23. Hemsworth LM, Jongman EC, Coleman GJ. The Human–Horse Relationship: Identifying the Antecedents of Horse Owner Attitudes towards Horse Husbandry and Management Behaviour. Animals. 2021;11: 278. doi:10.3390/ani11020278

24. Potter SJ, Bamford NJ, Harris PA, Bailey SR. Prevalence of obesity and owners' perceptions of body condition in pleasure horses and ponies in south-eastern Australia. Aust Vet J. 2016;94: 427–432. doi:10.1111/avj.12506

25. Furtado T, Perkins E, Pinchbeck G, McGowan C, Watkins F, Christley R. Exploring horse owners' understanding of obese body condition and weight management in UK leisure horses. Equine Vet J. 2021;53: 752–762. doi:10.1111/evj.13360

26. Morrison PK, Harris PA, Maltin CA, Grove-White D, Barfoot CF, Argo CMG. Perceptions of Obesity and Management Practices in a UK Population of Leisure-Horse Owners and Managers. J Equine Vet Sci. 2017;53: 19–29. doi:10.1016/j.jevs.2017.01.006

27. Light N, Fernbach PM, Rabb N, Geana MV, Sloman SA. Knowledge overconfidence is associated with anti-consensus views on controversial scientific issues. Sci Adv. 2022 p. 38. Available: https://www.science.org

28. R Core Team. R: A language and environment for statistical computing. R Found Stat Comput Vienna Austria. 2021 [cited 9 Aug 2022]. Available: https://www.R-project.org/.

29. Sison CP, Glaz J. Simultaneous Confidence Intervals and Sample Size Determination for Multinomial Proportions. J Am Stat Assoc. 1995;90: 366–369. doi:10.2307/2291162

30. Pek J, Wong O, Wong ACM. How to Address Non-normality: A Taxonomy of Approaches, Reviewed, and Illustrated. Front Psychol. 2018;9: 2104. doi:10.3389/fpsyg.2018.02104

31. Tingley D, Yamamoto T, Hirose K, Imai K. mediation: R Package for Causal Mediation Analysis. J Stat Softw. 2014;59: 1–38. doi:10.18637/jss.v059.i05

32. Imai K, Tingley D, Keele L. A General Approach to Causal Mediation Analysis. Psychol Methods. 2010;15: 309–334.

33. Heiberger RM, Robbins NB. Design of Diverging Stacked Bar Charts for Likert Scales and Other Applications. J Stat Softw. 2014;57. doi:10.18637/jss.v057.i05

34. Taber KS. The Use of Cronbach's Alpha When Developing and Reporting Research Instruments in Science Education. Res Sci Educ. 2018;48: 1273–1296. doi:10.1007/s11165-016-9602-2

35. Carroll C, Huntington P. Body condition scoring and weight estimation of horses. 20, 41-45. Equine Vet J. 1988;20: 41–45.

36. Henneke D, Kreider J, Yeates B. Relationship between condition score,physical measurements and body fat percentage in mares. Equine Vet J. 1983;15: 371-372.

37. Wyse CA, McNie KA, Tannahil VJ, Murray JK, Love S. Prevalence of obesity in riding horses in Scotland. Vet Rec. 2008;162: 590–591. doi:10.1136/vr.162.18.590

38. Bushell R, Murray J. A survey of senior equine management: Owner practices and confidence. Livest Sci. 2016;186: 69–77. doi:10.1016/j.livsci.2015.04.024

39. Golding E, Neavyn Neita A, Walshe N, Hanlon A, Mulcahy G, Duggan V. Survey of the knowledge and perceptions of horse owners in Ireland of common clinical conditions and their impact. Equine Vet J. 2022. doi:10.1111/evj.13589

40. Harris PA, Bamford NJ, Bailey SR. Equine metabolic syndrome: Evolution of understanding over two decades: A personal perspective. Anim Prod Sci. 2020;60: 2103–2110. doi:10.1071/AN19386

41. Durham AE, Frank N, McGowan CM, Menzies-Gow NJ, Roelfsema E, Vervuert I, et al. ECEIM consensus statement on equine metabolic syndrome. J Vet Intern Med. 2019;33: 335–349. doi:10.1111/jvim.15423

42. Furtado T, Perkins E, Pinchbeck G, McGowan C, Watkins F, Christley R. Hidden in Plain Sight: Uncovering the Obesogenic Environment Surrounding the UK's Leisure Horses. Anthrozoos. 2021;34: 491–506. doi:10.1080/08927936.2021.1914431

43. Cameron A, Harris P, Longland A, Horseman S, Hockenhull J. UK Horse Carers' Experiences of Restricting Grazing When Aiming to Prevent Health Issues in Their Horses. J Equine Vet Sci. 2021;104. doi:10.1016/j.jevs.2021.103685

44. Scheibe KM, Streich WJ. Annual Rhythm of Body Weight in Przewalski Horses (Equus ferus przewalskii). Biol Rhythm Res. 2003;34: 383–395. doi:10.1076/brhm.34.4.383.26227

45. Harris PA, Ellis AD, Fradinho MJ, Jansson A, Julliand V, Luthersson N, et al. Review: Feeding conserved forage to horses: Recent advances and recommendations. Animal. 2017;11: 958–967. doi:10.1017/S1751731116002469

46. Harris PA, Nelson S, Carslake HB, Argo CMG, Wolf R, Fabri FB, et al. Comparison of NIRS and Wet Chemistry Methods for the Nutritional Analysis of Haylages for Horses. J Equine Vet Sci. 2018;71: 13–20. doi:10.1016/j.jevs.2018.08.013

47. Moore-Colyer MJS, Lumbis K, Longland A, Harris P. The Effect of Five Different Wetting Treatments on the Nutrient Content and Microbial Concentration in Hay for Horses. PLOS ONE. 2014;9: e114079-.

48. Longland AC, Barfoot C, Harris PA. Effect of period, water temperature and agitation on loss of water-soluble carbohydrates and protein from grass hay: Implications for equine feeding management. Vet Rec. 2014;174: 68. doi:10.1136/vr.101820

49. Carslake HB, Argo CMG, Pinchbeck GL, Dugdale AHA, McGowan CM. Insulinaemic and glycaemic responses to three forages in ponies. Vet J. 2018;235: 83–89. doi:10.1016/j.tvjl.2018.03.008

50. Charles Abraham, Sarah Denford. Design, Implementation, and Evaluation of Behaviour Change Interventions: A Ten-Task Guide. In: Martin S. Hagger, Linda D. Cameron, Kyra Hamilton, Nelli Hankonen, Taru Lintunen, editors. The Handbook of Behavior Change. United Kingdom: Cambridge University Press; 2020.

51. Glanville C, Abraham C, Coleman G. Human Behaviour Change Interventions in Animal Care and Interactive Settings: A Review and Framework for Design and Evaluation. Animals. 2020;10: 2333. doi:10.3390/ani10122333

52. Spitznagel MB, Patrick K, Gober MW, Carlson MD, Gardner M, Shaw KK, et al. Relationships among owner consideration of euthanasia, caregiver burden, and treatment satisfaction in canine osteoarthritis. Vet J. 2022;286: 105868. doi:10.1016/j.tvjl.2022.105868

53. Mcelreath R. Statistical Rethinking 2 A Bayesian Course with Examples in R and Stan Second Edition. 2019.

Supporting information

S1 File. Table S1, Fig S1 and Supporting Information S1- additional respondent demographics and management practices information.

S2 File. Survey questionnaire.

S1 Supporting information. Code used for survey analysis. https://github.com/Ashley-B-Ward/AW_Survey_Analysis_2023

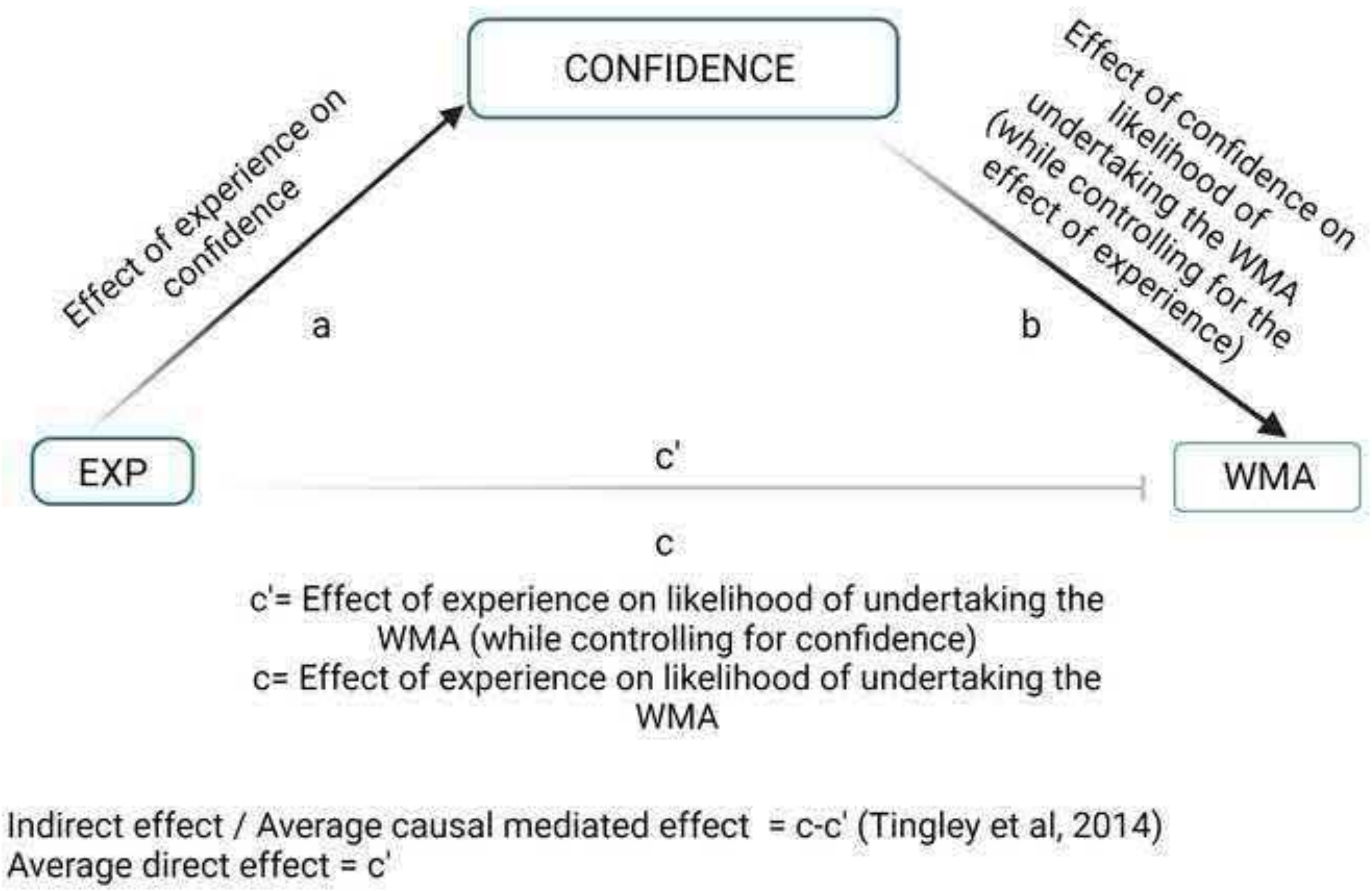
CONFIDENCE
Effect of experience on confidence
a
Effect of confidence on likelihood of undertaking the WMA (while controlling for the effect of experience)
b
EXP
c'
WMA
c
c'= Effect of experience on likelihood of undertaking the WMA (while controlling for confidence)
c= Effect of experience on likelihood of undertaking the WMA
Indirect effect / Average causal mediated effect = c-c' (Tingley et al, 2014)
Average direct effect = c'

Fig 2

Click here to access/download;Figure;Fig 2.tif

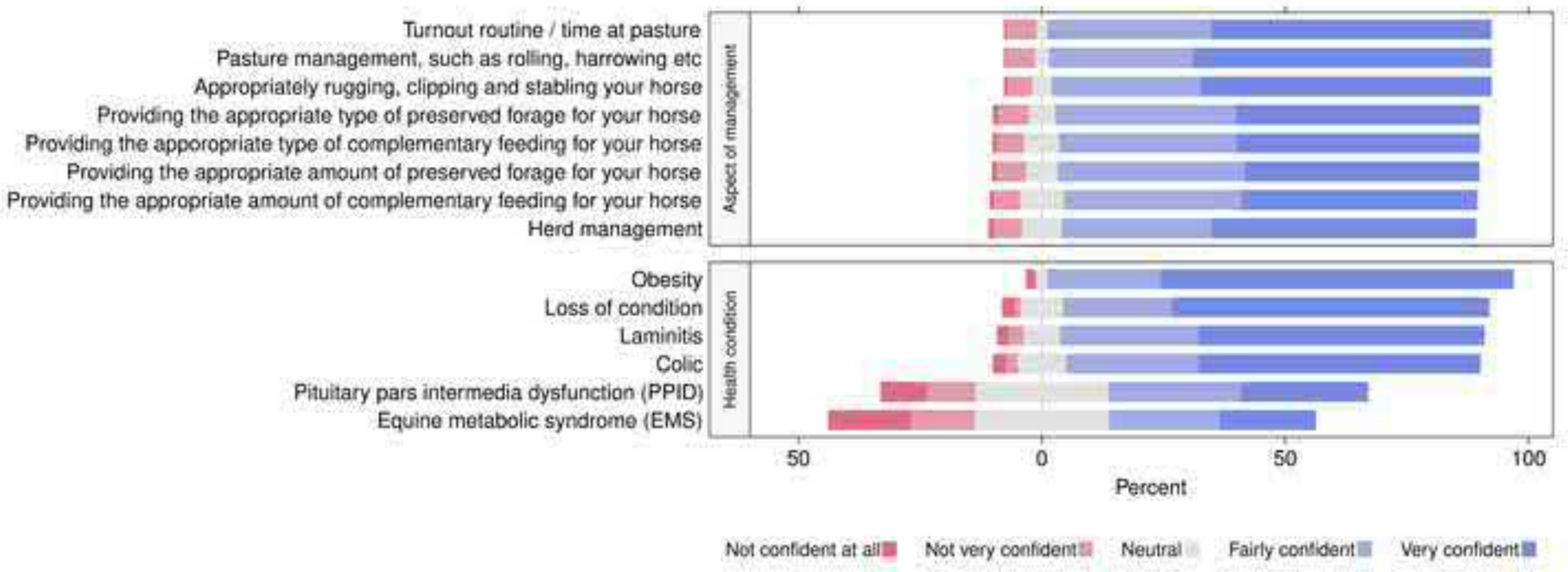

Fig 3

Click here to access/download;Figure;Fig 3.tif

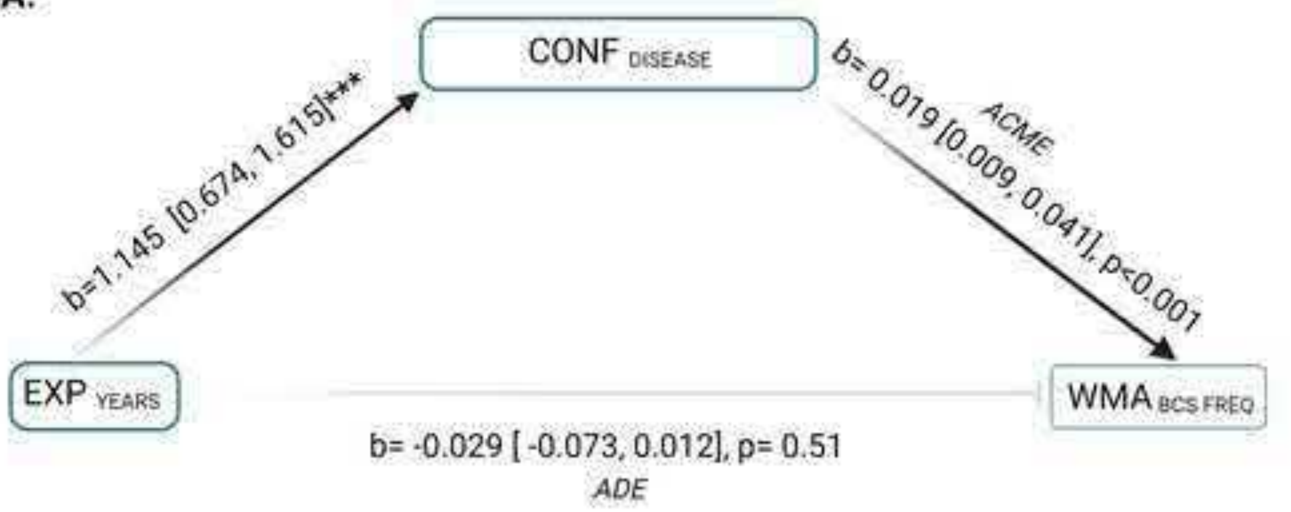

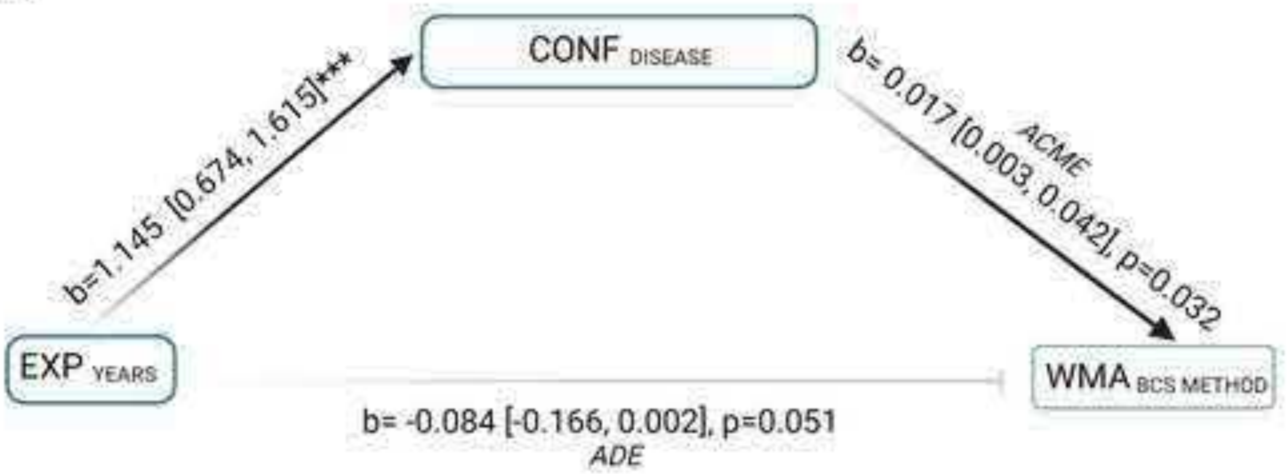

Fig 4

Click here to access/download;Figure;Fig 4.tif

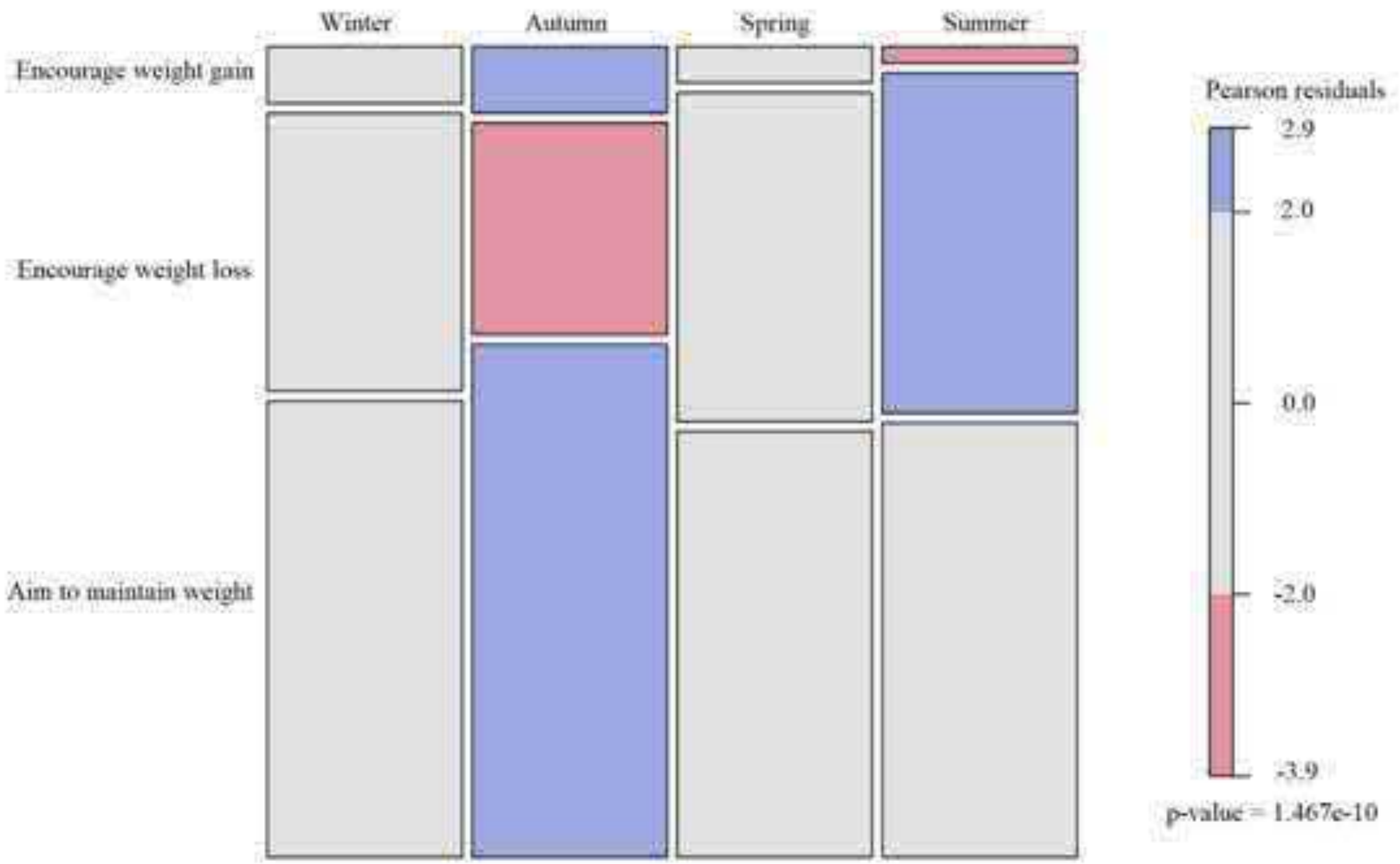

Fig 5

Click here to access/download;Figure;Fig 5.tif

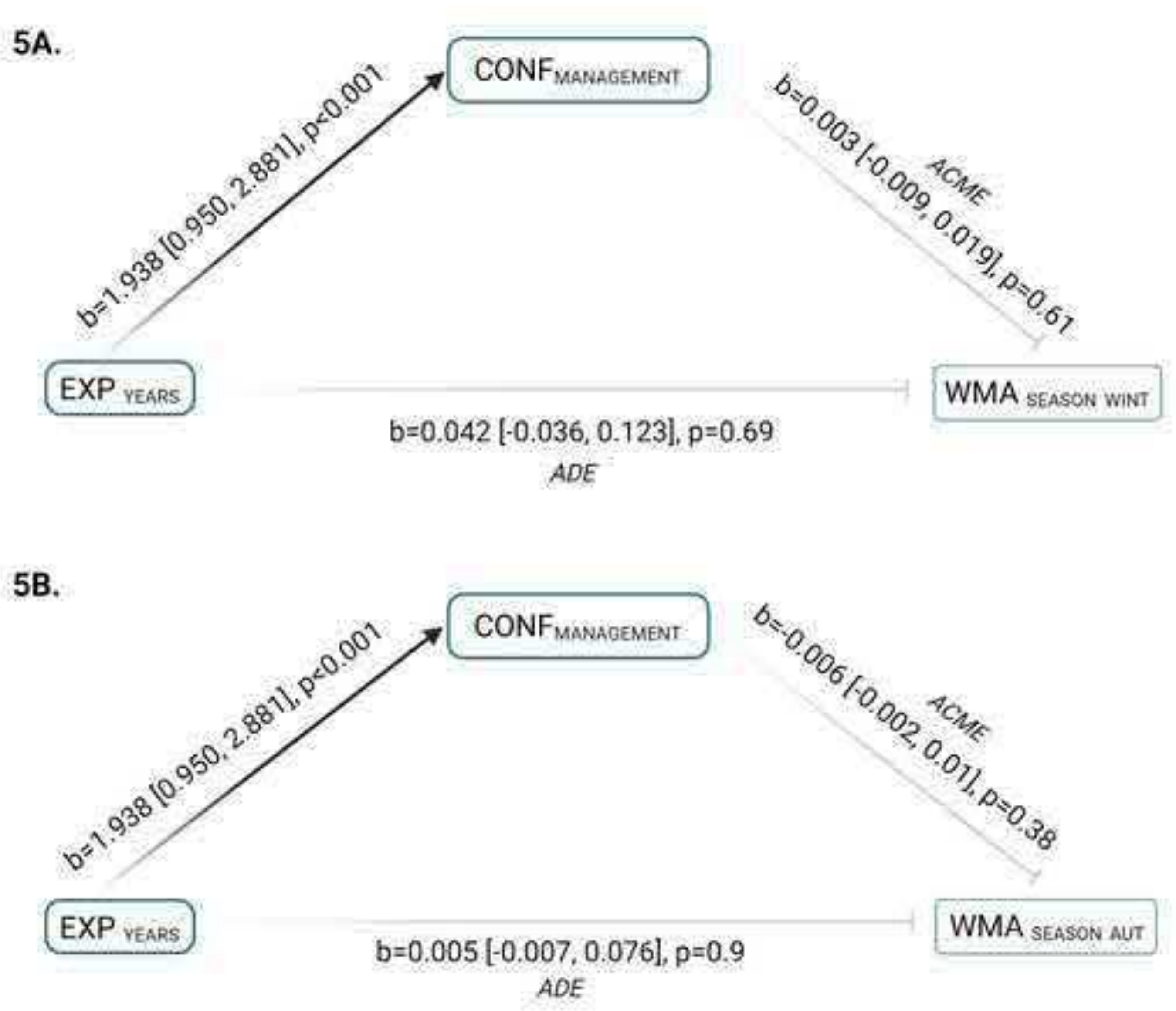

Fig 6

Click here to access/download;Figure;Fig 6.tif

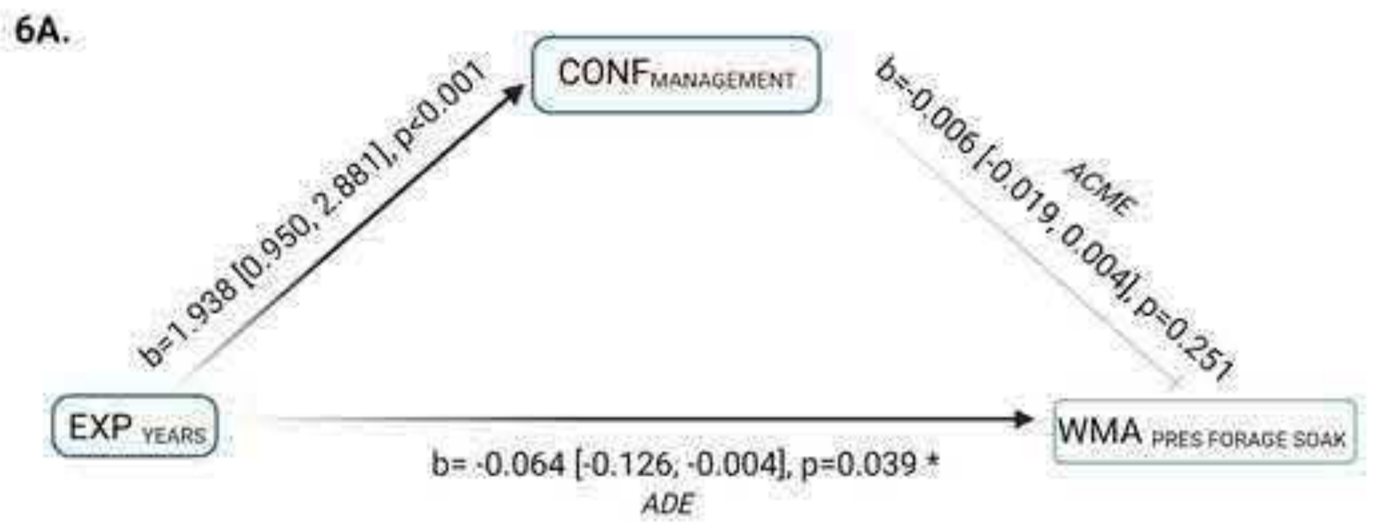

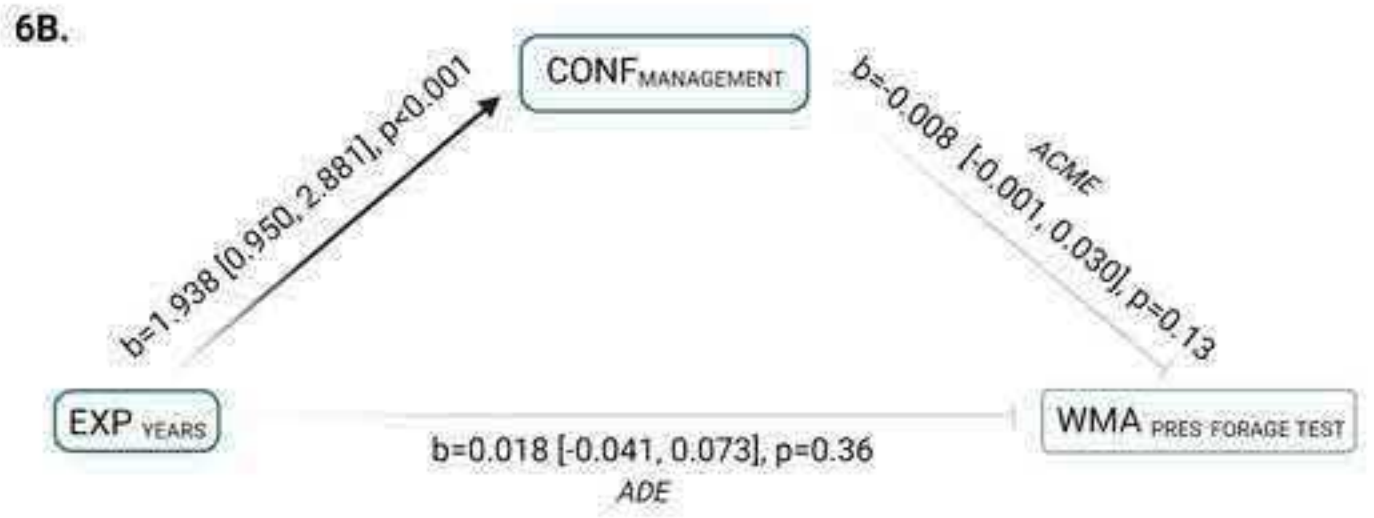

Fig 7

Click here to access/download;Figure;Fig 7.tif

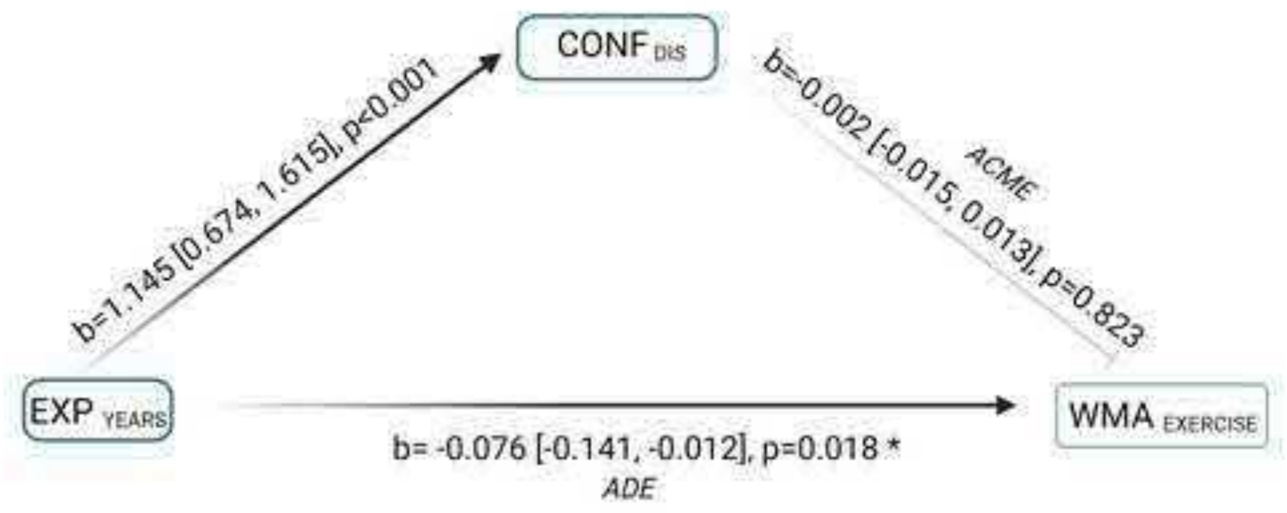

Supporting Information (S1)

Click here to access/download
Supporting Information
S1_File.docx

Supporting Information (S2)

Click here to access/download
Supporting Information
S2_File.docx

Fig1 publication licence from BioRender

Click here to access/download
Other
Fig1 _Publication License Apr-10-2023.pdf

Fig3 publication licence from BioRender

Click here to access/download

Other

Fig3_Publication License Apr-10-2023 (2).pdf

Fig5 publication licence from BioRender

Click here to access/download
Other
Fig5_Publication License Apr-10-2023 (1).pdf

Fig6 publication licence from BioRender

Click here to access/download
Other
Fig6_Publication License Apr-10-2023 (3).pdf

Fig7 publication licence from BioRender

Click here to access/download

Other

Fig7_Publication License Apr-10-2023 (4).pdf

Ethical approval document

Click here to access/download
Other
renamed_f9241.doc

S1 Supporting information: GitHub repository with R code for survey analysis

Click here to download Data Review URL http://github.com/Ashley-B-Ward/AW_Survey_Analysis_2023
